# Autotrophic growth of *Thermus* sp. PS18 and its genomic determinants shed light on the autotrophic lifestyle and its evolution in the *Thermaceae* family

**DOI:** 10.3389/fmicb.2026.1769897

**Published:** 2026-03-12

**Authors:** Tatyana G. Sokolova, Alexander G. Elcheninov, Galina B. Slobodkina, Ekaterina A. Provotorova, Maria I. Zvereva, Alexander V. Lebedinsky, Nikolai A. Chernyh

**Affiliations:** 1 Winogradsky Institute of Microbiology, Federal Research Center of Biotechnology, Russian Academy of Sciences, Moscow, Russia; 2 Department of Chemistry, M.V. Lomonosov Moscow State University, Moscow, Russia

**Keywords:** autotrophy, genomic analysis, bacterial evolution, HGT, polyploidy, heterozygosity, *Thermus*, *Deinococcota*

## Abstract

A thermophilic bacterial strain PS18 was isolated from a hot spring on Kunashir Island under aerobic autotrophic conditions and identified as *Thermus brevis*. Autotrophy has never been demonstrated in representatives of the *Thermaceae* family or the entire *Deinococcota* phylum. *T. brevis* PS18, in addition to the capacity for heterotrophic growth, showed sustainable autotrophic growth on thiosulfate in aerobic conditions. It could also grow anaerobically with formate and nitrate. Autotrophic growth of *T. brevis* PS18 was characterized in growth experiments, as well as by radioisotopic, genomic and proteomic analyses, and the leading role of the CBB cycle was demonstrated. Analysis of *Deinococcota* genomes available in GenBank revealed CBB cycle determinants in 24 species of the *Thermaceae* family. We further demonstrated autotrophic growth of *T. caldilimi* YIM 78456^T^, one of the carriers of CBB cycle determinants, and isolated two more autotrophically growing *Thermus* strains, *T. oshimai* Uz8 and *T. scotoductus* Uz79, from hot spring samples. These results indicate widespread occurrence of autotrophic CO__2__ assimilation in representatives of the *Thermaceae* family, and suggest that they may be among primary producers in microbial communities of natural and anthropogenic thermal environments. Bioinformatic insight into the evolution of the autotrophic capacity in *Thermaceae* revealed remarkable lateral mobility of autotrophy key determinants in this family, which we explain in terms of our hypothesis of inheritance of facultative characters by gene loss and reacquisition from the pangenome. We also present bioinformatic evidence that in *Thermaceae* the (re)acquisition mechanism may involve heterozygous stage sustainable over generations.

## Introduction

Climate change associated with greenhouse gas emissions into the atmosphere is one of the most important challenges facing humanity. Carbon dioxide levels in 2023 were 151% of pre-industrial levels. This change in the chemical composition of the atmosphere leads to changes in weather patterns and natural imbalances, which poses a huge threat to humans and all life on Earth (World Meteorological Organization, 2023). Autotrophic microorganisms serve as a natural biofilter and reduce the emission of this greenhouse gas; therefore, expanding our knowledge of autotrophic prokaryotes is of evident importance. In this paper, we present data on the previously unknown autotrophic metabolism of representatives of the genus *Thermus* in the phylum *Deinococcota*. Representatives of the genus *Thermus*, extreme thermophiles widely known as producers of thermostable polymerases Taq and Tth, which have revolutionized molecular biology and medicine, were first isolated and described in 1969 (Brock and Freeze, 1969) and have since then been considered chemoorganoheterotrophs using diverse organic compounds (Nobre et al., 1996); later, some species were shown to grow chemolithoheterotrophically (Skirnisdottir et al., 2001; Zhou et al., 2020). It has been noted that some species of the genus *Thermus* contain a complete set of genes of the CBB cycle (Müller et al., 2016; Khan et al., 2024). The occurrence of CBB cycle genes in *Thermus* MAGs assembled from hot spring metagenomes has also been reported, and their possible contribution to primary production has tentatively been supposed based on indirect evidence (DeCastro et al., 2021; Colman et al., 2024). However, autotrophic growth of *Thermus* species has never been demonstrated. Representatives of the genus *Thermus* are widespread in neutral and alkaline terrestrial and submarine hot springs all over the world, as well as in anthropogenic thermal habitats (Nobre et al., 1996; Wilpiszeski et al., 2019; Saghatelyan et al., 2021; Colman et al., 2024; Keller et al., 2026). Thus, the knowledge on the autotrophic capacities of *Thermus* spp., shared in this communication, is to expand the understanding of the processes of carbon dioxide fixation in thermal habitats.

## Materials and methods

### Origins of the strains

Strain PS18 was isolated from a hot spring located on the shore of Lake Kipyashchee, Kunashir Island, Russia (43.864997 ° N, 145.500474 ° E). Samples were collected in August 2014. The temperature at the sampling site was 73 °C, and the pH ranged from 8.0 to 8.5. Strains Uz79 and Uz8 were isolated from water and sediment samples collected from a stream formed by the discharge of artesian groundwater in the Navoi region, Uzbekistan (40.16226 ° N, 66.01274 ° E). Specifically, strain Uz79 was isolated from a puddle beneath the groundwater discharge pipe, whereas strain Uz8 was obtained from a sample collected about 10 m downstream. Sampling was carried out in October 2022. Temperatures at the sampling sites were 60 °C and 54 °C for Uz79 and Uz8, respectively, and the pH was 6.5. *Thermus caldilimi* YIM 78456^T^ (=KCTC 52,948^T^; Li et al., 2019) was obtained from the Korean Collection for Type Cultures.

### Media and cultivation conditions

For enrichment, isolation, and cultivation of microorganisms, liquid mineral medium of the following composition (g L^−1^) was used: NH_4_Cl, 1.0; MgCl_2_·2H_2_O, 0.33; CaCl_2_·6H_2_O, 0.10; KCl, 0.33; KH_2_PO_4_, 0.50; NaHCO_3_, 1.0. The medium was supplemented with 1 mL L^−1^ of trace elements solution (Pfennig and Lippert, 1966) and 1 mL L^−1^ of vitamin solution (Wolin et al., 1963).

Unless otherwise stated, strains PS18, Uz8, and *T. caldilimi* YIM 78456^T^ were cultivated in rubber-stoppered 50-mL or 2-L flasks at a gas-to-liquid phase ratio of 4:1 without shaking. For heterotrophic growth, yeast extract (0.5 g L^−1^) was used as the substrate, and the gas phase was air. Autotrophic cultivation was performed with Na_2_S_2_O_3_ (10 mM) as the electron donor and a gas phase composed of air supplemented with 10% CO_2_ (v/v). For cultivation with carbon monoxide or hydrogen, these gases were added to air at a concentration of 10% (v/v) in the gas phase. Anaerobic cultivation was carried out in medium supplemented with Na_2_S·9H_2_O (0.3 g L^−1^), sodium formate (30 mM), and NaNO_3_ (10 mM), with an Ar:CO_2_ (9:1, v/v) gas phase. Cultivation temperatures were 65 °C for strains PS18 and Uz8 and 55 °C for *T. caldilimi* YIM 78456^T^.

Strain Uz79 was cultivated in 17-mL Hungate tubes containing 10 mL of liquid medium with a gas phase of air and CO_2_ (1:1, v/v). For autotrophic growth, elemental sulfur (5 g L^−1^) or sodium thiosulfate (10 mM) was used as the electron donor. For heterotrophic growth, the medium was supplemented with yeast extract, peptone, and glucose (2.0 g L^−1^ each). During cultivation with hydrogen as the energy source, the gas phase consisted of H_2_:CO_2_:O_2_ (8:1:1, v/v). Cultivations were performed without shaking at 60 °C.

### Microscopy

Cell morphology was examined under a 1,000 × phase-contrast light microscope CX41RF (Olympus). The whole cells, negatively stained with 2% (w/v) phosphotungstic acid, as well as ultrathin sections of the cells, stained by uranyl-acetate and lead citrate, were visualized using a JEM-100 transmission electron microscope (JEOL) at the UNIQEM Collection Core Facility, Research Center of Biotechnology of the Russian Academy of Sciences.

### Analytical methods

Bacterial growth was estimated by direct cell counting using phase-contrast light microscope Olympus CX43. The biomass protein was determined by the method of Lowry et al. (1951). Thiosulfate in batch cultures was determined by iodometric titration (Listov and Arzamastsev, 1987) after acidification of the samples with acetic acid to a pH of 5.0. Formate, nitrate, and nitrite were analyzed on a Capel-205 (Lumex, Russia) new generation capillary electrophoresis system. Formate was determined using a background electrolyte based on cetyltrimethylammonium bromide, phosphate buffer, and isopropyl alcohol. The separation was carried out in a capillary with a length of 75 cm and an inner diameter of 50 μm at a voltage of −25 kV. The detection was performed spectrophotometrically at a wavelength of 190 nm (Litti et al., 2022). For determination of nitrate and nitrite, electrophoresis was performed in untreated fused-silica capillaries of 60 cm length (effective length, 50 cm) and 75 μm internal diameter. The capillary was held at 20 °C and the applied voltage was +13 kV for cations or −17 kV for anions Identification and quantitation of the analyzed anions were performed by measuring UV absorption at 254 nm (Popova et al., 2023). Concentrations of N__2__ and N__2__O in the gas phase were determined using a Kristall 5000.2 gas chromatograph (Khromatek, Russia). The sorbents used for determination of N__2__ and N__2__O were zeolite NaX 60/80 mesh and Porapak Q 80/100 mesh. The steel column was 3 m long with 2-mm inner diameter; the column temperature was 60 °C, injector temperature was 200 °C, katharometer temperature was 200 °C, and the flow of the carrier gas (argon) was 25 mL · min^−1^.

### Radioisotopic analysis of autotrophic CO_2_ fixation by strain PS18

Strain PS18 was grown aerobically, autotrophically in the mineral medium with ${\text{HCO}}_{3}^{-}$ as the carbon source on thiosulfate and heterotrophically in mineral medium supplemented with 500 mg L^−1^ of yeast extract. The cells were collected by centrifugation and washed with growth medium lacking carbon and nitrogen sources. Then the cells were resuspended in the same medium, and the suspensions were supplemented with 2 mM NaHCO_3_ and 2 mM Na__2__S__2__O_3_. The optical density (OD_600_) of the autotrophically grown cell suspension was 0.220, the protein content was 0.14 mg · mL^−1^. The OD_600_ of the heterotrophically grown cell suspension was 0.275, the protein content was 0.16 mg · mL^−1^. The resulting suspensions were put as 5 mL aliquots into 23-mL flasks in duplicate; the gas phase was air. After this, H^14^CO_3_-was added to the flasks. The activity of the label in the stock solution was 28 × 10^4^ Bq per mL (5.6 × 10^4^ Bq per mL in the resulting mixture). The flasks were incubated on a shaker at a temperature of 60 °C for 6 h; samples were taken every 1.5–2 h. After incubation, the samples were fixed with 1 mL of 0.1 N NaOH solution. Each series of measurements had abiotic controls, which were samples from flasks to which NaOH solution had been added at the onset of incubation. The cell suspensions were filtered; the filters were dried and placed in vials with scintillation liquid. Radioactivity of the products was measured on a TRI-CarbTR 2400 liquid scintillation counter (Packard, Conroe, TX, USA). The rates of CO__2__ fixation were calculated as described by Pimenov and Bonch-Osmolovskaya (2006).

### Strains PS18 and Uz79 genome sequencing and assembly

For genome sequencing, strain PS18 was grown aerobically autotrophically on mineral medium with thiosulfate as electron donor and energy source. Strain Uz79 was grown aerobically on mineral medium supplemented with yeast extract, peptone, and glucose (2.0 g L^−1^ each).

Genomic DNA was isolated from cells using FastDNA SPIN Kit for Soil following the manufacturer's recommendations (http://www.mpbio.com).

The fragment libraries were prepared from 0.5 μg of genomic DNA with an NEBNext Ultra DNA Library Preparation Kit (New England Biolabs, Ipswich, MA, USA) according to the manufacturer's instructions to obtain a mean library size of 700 bp. The resulting libraries were sequenced on a MiSeq Personal Sequencing System (Illumina, San Diego, CA, USA) using a 2 × 10^1^-bp paired-end read cartridge. The obtained Illumina reads were filtered and trimmed using CLC Genomics Workbench 10.0 (Qiagen). Nanopore sequencing (Oxford Nanopore Technology, Oxford, UK) for strain PS18 was performed as described earlier (Zayulina et al., 2024).

Trimmed short high quality reads as well as long reads were taken for *de novo* hybrid assembly using Unicycler v 0.4.9 with default parameters (Wick et al., 2017). Assembly statistics was checked using Quast v 5.0.2 with default parameters (Mikheenko et al., 2018). Completeness and contamination were estimated using CheckM v.1.2.2 with *Bacteria* marker set (Parks et al., 2015).

### Functional genome analyses

Primary genome annotation of strains PS18 and Uz79 was performed by NCBI PGAP v.6.2 (Tatusova et al., 2016) during the process of genome submission to NCBI submission portal. Additional annotations aimed to improve predictions of protein function were performed using RAST web server (Overbeek et al., 2014) and manual curation of key metabolic genes. Determinants of the metabolic pathways that were of special interest in this work (CBB cycle, roTCA cycle, denitrification, sulfur compound oxidation) were searched in strain PS18 genome using KEGG Pathway Database (Kanehisa et al., 2023) and manual curation that employed blastp searches, the queries being either strain PS18 proteins (blastp in NCBI nr) or reference proteins (blastp in PS18 genome) taken from relevant papers (Friedrich et al., 2000, 2001; Murugapiran et al., 2013; Greening et al., 2016; Jeoung et al., 2019; Sánchez-Andrea et al., 2020).

Signal peptides and transmembrane helices in proteins were identified using the Phobius server (Käll et al., 2004). Non-classically secreted proteins were predicted using the SecretomeP-2.0 server (Bendtsen et al., 2004).

Searches for determinants of the CBB cycle and lithotrophy in *Thermaceae* spp. genomes were performed by blastp (Altschul et al., 1990) in the corresponding genomes present in NCBI nr. The queries were relevant proteins that we revealed to be encoded in the strain PS18 genome (Supplementary Tables 1, 4; note that the role of these proteins and thus their adequacy as queries were confirmed in this work by proteomic analysis, see Results and discussion section). If necessary, queries taken from relevant papers (Friedrich et al., 2000, 2001; Murugapiran et al., 2013; Greening et al., 2016; Jeoung et al., 2019) were also used. In cases where it was of particular importance to fully trust the phylotaxonomic affiliation of blast hits to uncultured subjects, we additionally performed tblastn searches in representative genomes of GTDB RS220 (Parks et al., 2026) downloaded from GTDB repository to create a local database for tblastn from NCBI-BLAST+ package (Camacho et al., 2009).

### Phylogenetic analyses and genome-based comparisons

Average nucleotide identities (ANI) were calculated using the Pairwise ANI tool at IMG system (Chen et al., 2023) or ANI calculator (Yoon et al., 2017). Digital DNA-DNA hybridization (dDDH) values were measured using GGDC v.3.0, either stand-alone or within TYGS (Meier-Kolthoff et al., 2022). Average amino acid identity (AAI) values were calculated from RAST/SEED sequence-based comparison tables using an *ad hoc* written script.

The phylogenetic analysis was based on the bac120 set of conserved proteins (Parks et al., 2017). The protein sequences were identified in genomes of *Thermaceae* family defined as species representatives in GTDB RS207 and were aligned with the GTDB-tk v2.1.0 (Chaumeil et al., 2019). The full alignment was used for phylogenetic tree construction with the RAxML v.8.2.12 (Stamatakis, 2014) with the PROTGAMMAILG model of amino acid substitution. Node support values were from 1,000 rapid bootstrap replications. Protein sequences for phylogenetic analysis of RuBisCO large subunit (CbbL) were selected and treated as described earlier (Maltseva et al., 2024) with some modifications: thresholds for cd-hit of 0.97, 0.9, 0.8 and 0.8 were used for CbbL of RuBisCO types I, II, III, and IV, respectively. The phylogenetic tree was constructed using IQtree v.2.3.5 (Minh et al., 2020) with LG+I+G4 model and 1,000 ultrafast bootstrap replications (Hoang et al., 2018). The trees were visualized and decorated using iTOL v.6 (Letunic and Bork, 2024).

Maximum Likelihood phylogenetic trees of RuBisCO large subunits of *Thermaceae* spp. type strains and of CISM catalytic subunits were constructed with Mega X. The bootstrap procedure with 1,000 replicates was used (Jones et al., 1992; Kumar et al., 2018).

### Strain PS18 comparative proteome analysis

For comparative proteomic analysis, strain PS18 was grown at 65 °C in three medium variants, each in three biological replicates, in 2-L bottles containing 600 mL of medium. The medium variants were as follows: aerobic mineral medium with 10 mM Na__2__S__2__O_3_, aerobic mineral medium supplemented with yeast extract (0.5 g L^−1^), and anaerobic mineral medium with sodium formate (30 mM) and NaNO_3_ (10 mM). The gas phase was air: CO__2__ (9:1 v/v) or N__2__:CO__2__ (9:1 v/v). Cells were collected by centrifugation at the end of the exponential growth phase.

The origins of chemicals used in further procedures are described in Supplementary Text 1. Cell lysis, reduction, alkylation, and digestion of proteins, and SCX StageTip fractionation were performed as described previously (Frolov et al., 2019).

NanoLC-MS/MS analysis was performed as follows. Peptide fractions were suspended in loading buffer (2% acetonitrile, 0.1% trifluoroacetic acid) and sonicated for 2 min in ultrasonic water bath (Elma, Elmasonic S100) before nanoLC-MS/MS analysis. Approximately 1 μg of peptides was loaded for 2 h gradient. Peptides were separated on a 25-cm 75-μm inner diameter column packed in-house with Aeris Peptide XB-C16 2.6 μm resin (Phenomenex). Reverse-phase chromatography was performed with an Ultimate 3000 Nano LC System (Thermo Fisher Scientific), which was coupled to the Q Exactive HF mass spectrometer (Thermo Fisher Scientific) via a nanoelectrospray source (Thermo Fisher Scientific). Peptides were loaded in buffer A (0.2% (v/v) formic acid) and eluted with a linear 120-min gradient of 4–55% buffer B (0.1% (v/v) formic acid, 80% (v/v) acetonitrile) at a flow rate of 350 nL min^−1^. After each gradient, the column was washed with 95% buffer B for 5 min and reequilibrated with buffer A for 5 min. Column temperature was kept at 40 °C. MS data were acquired with an automatic switch between a full scan and up to 15 data-dependent MS/MS scans (topN method). Target value for the full scan MS spectra was 3 × 10^6^ in the 300–1,200 m/z range with a maximum injection time of 60 ms and a resolution of 60,000. Isolation of precursors was performed with a 1.4 m/z window and a fixed first mass of 100.0 m/z. Precursors were fragmented by higher-energy C-trap dissociation with a normalized collision energy of 28 eV. MS/MS scans were acquired at a resolution of 15,000 at m/z 400 with an iron target value of 1 × 10^5^ in the 200–2,000 m/z range with a maximum injection time of 30 ms. Repeat sequencing of peptides was minimized by excluding the selected peptide candidates for 30 s.

Data analysis was performed as described previously (Frolov et al., 2019).

## Results and discussion

### Enrichment and isolation of strain PS18

A sample of water and mud from a hot spring on Kunashir Island, Russia (see section Materials and methods for details), collected in a sterile Falcon tube and transported to the laboratory, was used to inoculate (10% v/v) 10 mL of the mineral medium under an air and CO (9:1) mixture in 50-mL bottles. The bottles were incubated at 65 °C, and after 3 days growth of various rod-shaped cells occurred. After several transfers in the same medium, the culture was dominated by a particular morphotype of straight short rods. The pure culture of strain PS18 was isolated on the same medium by serial 10-fold dilution technique.

**Figure 1:**
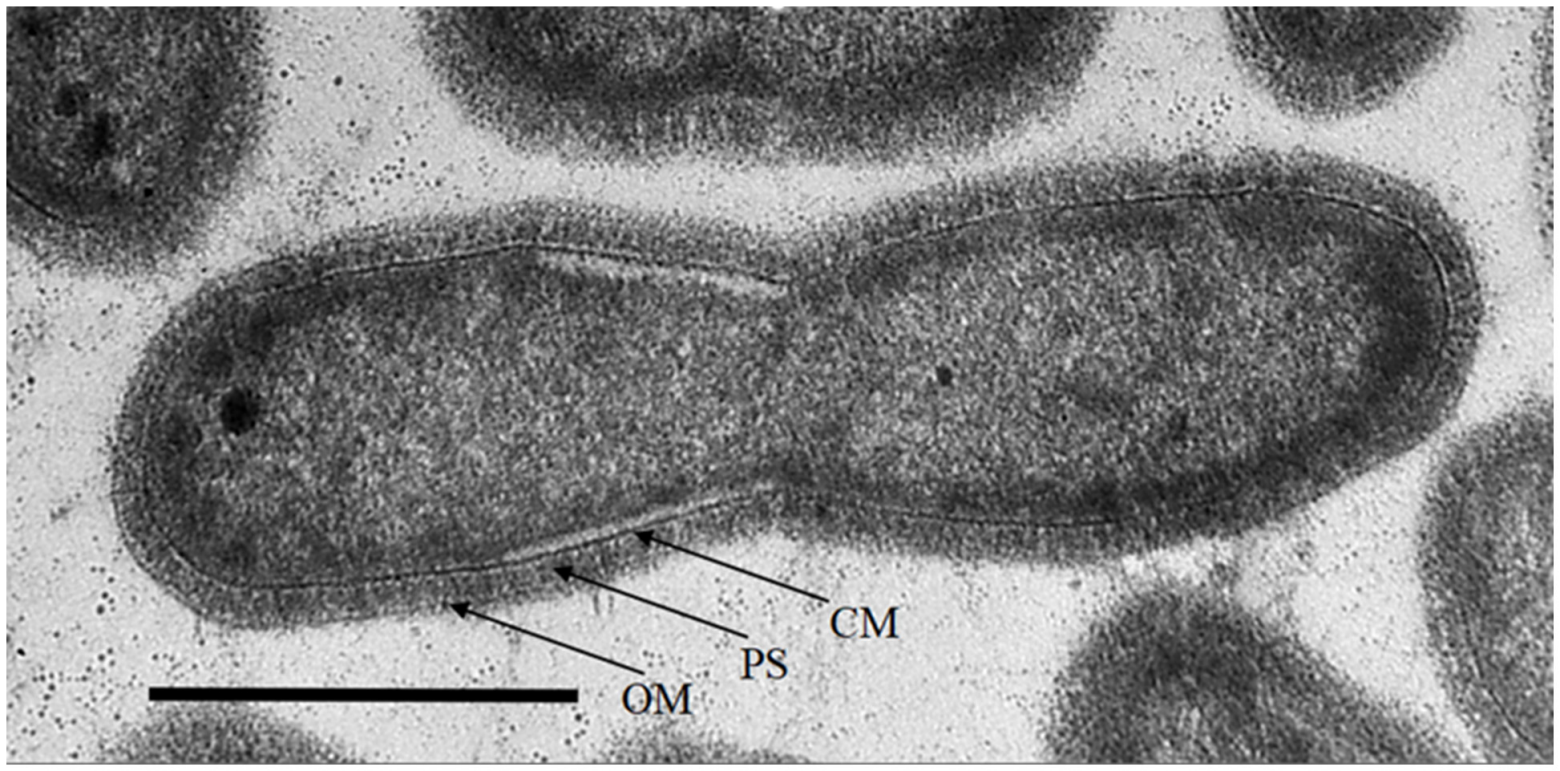
Transmission electron microscopy of strain PS18 cell thin section, showing binary fission. Bar, 0.5 μm. OM, outer membrane; PS, periplasmic space; CM, cytoplasmic membrane.

Strain PS18 cells were straight short non-motile rods, 0.46 × 0.9–1 μm. Ultrathin sections revealed cell envelope structure of the Gram-negative type (Figure 1).

Sequencing of the 16S rRNA gene by the Sanger method and BLAST search for closest homologs within NCBI core_nt database suggested affiliation of strain PS18 with the genus *Thermus* (91–98% identity with homologs from *Thermus* spp.; closer relatives were not available at that moment of our investigation).

Strain *Thermus* sp. PS18 was deposited in Collection of Unique and Extremophilic Microorganisms (UNIQEM) under the designation UQM 41925 and in All-Russian Collection of Microorganisms (VKM) under the number B-3909.

### Autotrophic growth of *Thermus* sp. PS18

**Figure 2:**
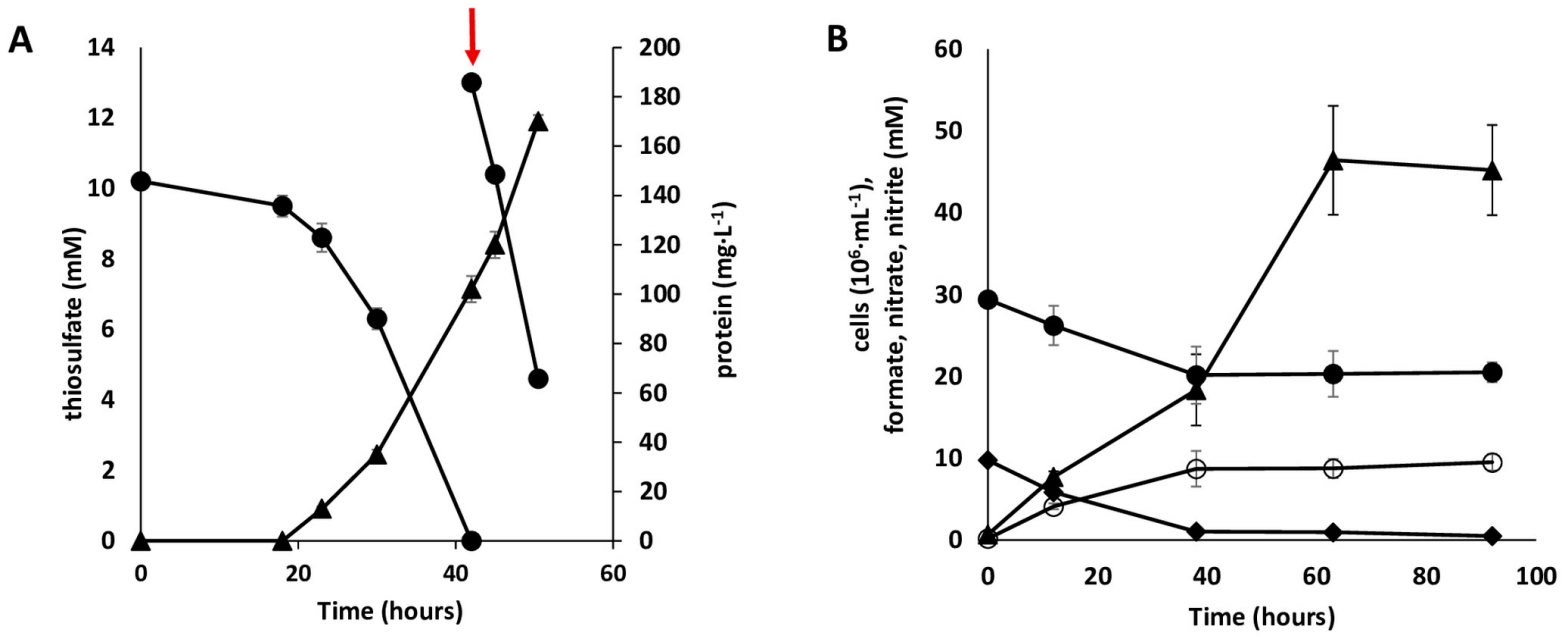
**(A)** Aerobic thiosulfate oxidation-dependent growth of *Thermus* sp. PS18 in mineral medium; circles, thiosulfate; triangles, protein; the arrow indicates the moment of repeated thiosulfate addition. **(B)** Anaerobic growth of *Thermus* sp. PS18 in mineral medium due to formate oxidation coupled to nitrate reduction with nitrite formation; triangles, cells; filled circles, formate; diamonds, nitrate; open circles, nitrite. There were three replicates of each cultivation variant.

Despite the enrichment and isolation of strain PS18 with CO as the substrate, the growth on CO was hardly reproducible in further work. Weak growth on H__2__ was observed in first transfers on this substrate, but it was not sustainable in culture transfers. However, apart from good growth in aerobic heterotrophic conditions with yeast extract as the substrate, the strain showed sustainable autotrophic growth in multiple culture transfers in aerobic conditions on thiosulfate (Figure 2A) and in anaerobic conditions on formate with nitrate (Figure 2B).

The best electron donor for autotrophic growth of *Thermus* sp. PS18 was found to be thiosulfate. After 42 h of growth, thiosulfate (initial concentration of 10 mM) was completely oxidized and a decrease in pH was observed. After adjusting the pH to 7.0 and adding thiosulfate, its oxidation and cell growth continued (Figure 2A).

During autotrophic growth in anaerobic conditions with formate, nitrate was reduced to nitrite in almost equimolar quantities (Figure 2B); however, formation of trace amounts of nitrous oxide was detected.

### Radioisotopic analysis of autotrophic CO_2_ fixation by *Thermus* sp. PS18

**Figure 3:**
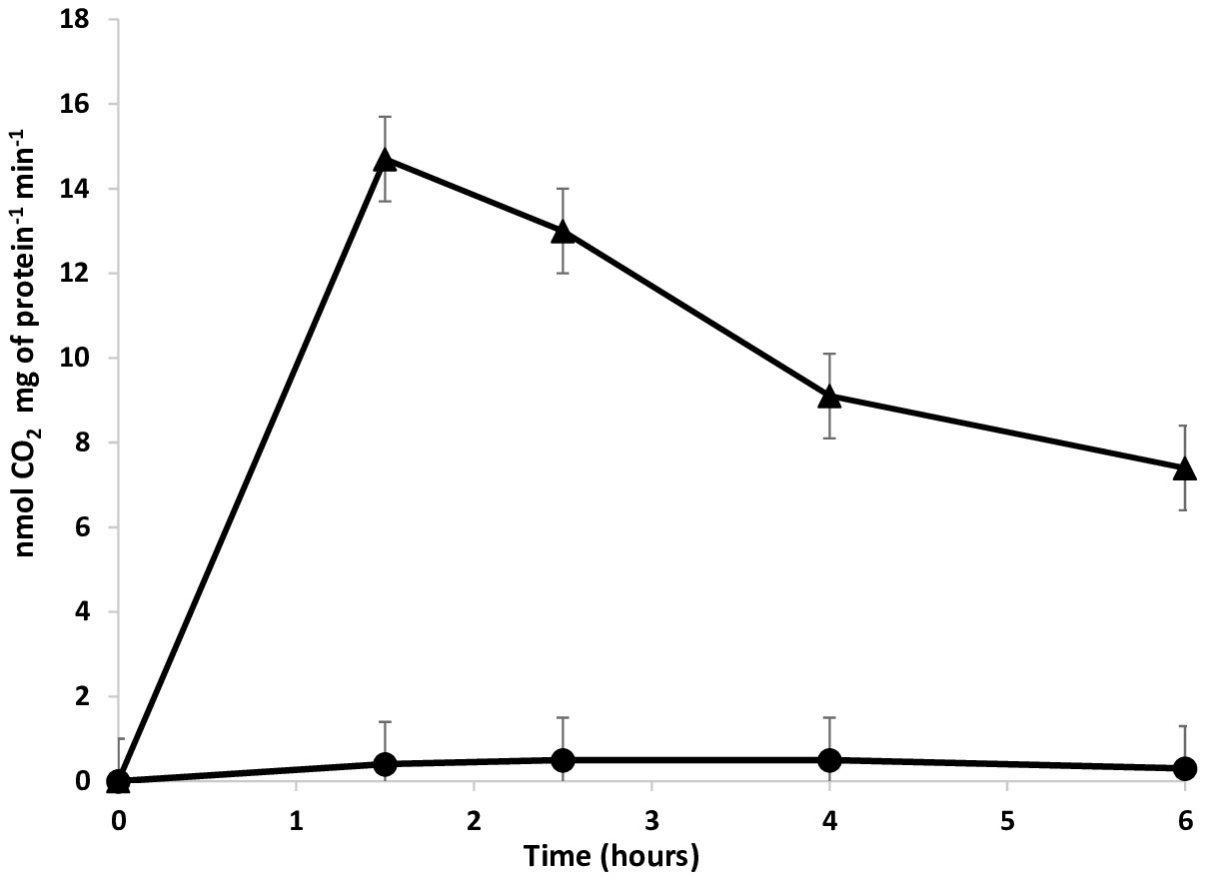
The rate of CO_2_ incorporation into cells resuspended in mineral medium with thiosulfate and without a nitrogen source. Cells were grown under autotrophic (mineral medium with thiosulfate, triangles) and heterotrophic conditions (mineral medium supplemented with 500 mg L^−1^ yeast extract, circles).

Radioisotopic analysis showed that suspensions of *Thermus* sp. PS18 cells grown under autotrophic conditions were capable of fixing carbon dioxide. In contrast, suspensions of cells grown under heterotrophic conditions did not have this ability (Figure 3). In autotrophically grown *Thermus* sp. PS18 cell suspensions, the per-cell carbon fixation activity was 1.2 × 10^−9^ μmol C · cell^−1^ · h^−1^, which is close to values measured for autotrophic bacteria growing by oxidizing reduced sulfur compounds. For example, the carbon fixation rate of “*Candidatus* Arcobacter sulfidicus” during chemolithoautotrophic growth on sulfide was 0.8 × 10^−9^ μmol C cell^−1^ h^−1^ in batch culture or 1.04 × 10^−9^ μmol C · cell^−1^ · h^−1^ in flow cultivation (Wirsen et al., 2002).

### General genomic features of *Thermus* sp. PS18

Complete genome assembly of *Thermus* sp. PS18, obtained using both Illumina and ONT reads, consists of a single circular chromosome (2,375,041 bp) as well as a circular plasmid (19,473 bp). Checking metrics using Quast showed that 99.9% of high accuracy Illumina reads were mapped to the hybrid genome assembly with average coverage depth of 1,047 (total length was 2,394,514 bp), indicating high accuracy of the assembly. According to the CheckM estimation (*Bacteria* marker set), completeness and contamination of the assembly were 100% and 0%, respectively. G+C content was 65.03%. The annotation revealed that the chromosome contained 2,494 protein-coding genes and 58 RNA genes including 48 tRNA genes, 6 rRNA genes (two copies of 5S, 16S, 23S) and 4 ncRNA. The plasmid contained 27 CDSs.

### Phylogenetic position of strain PS18

**Figure 4:**
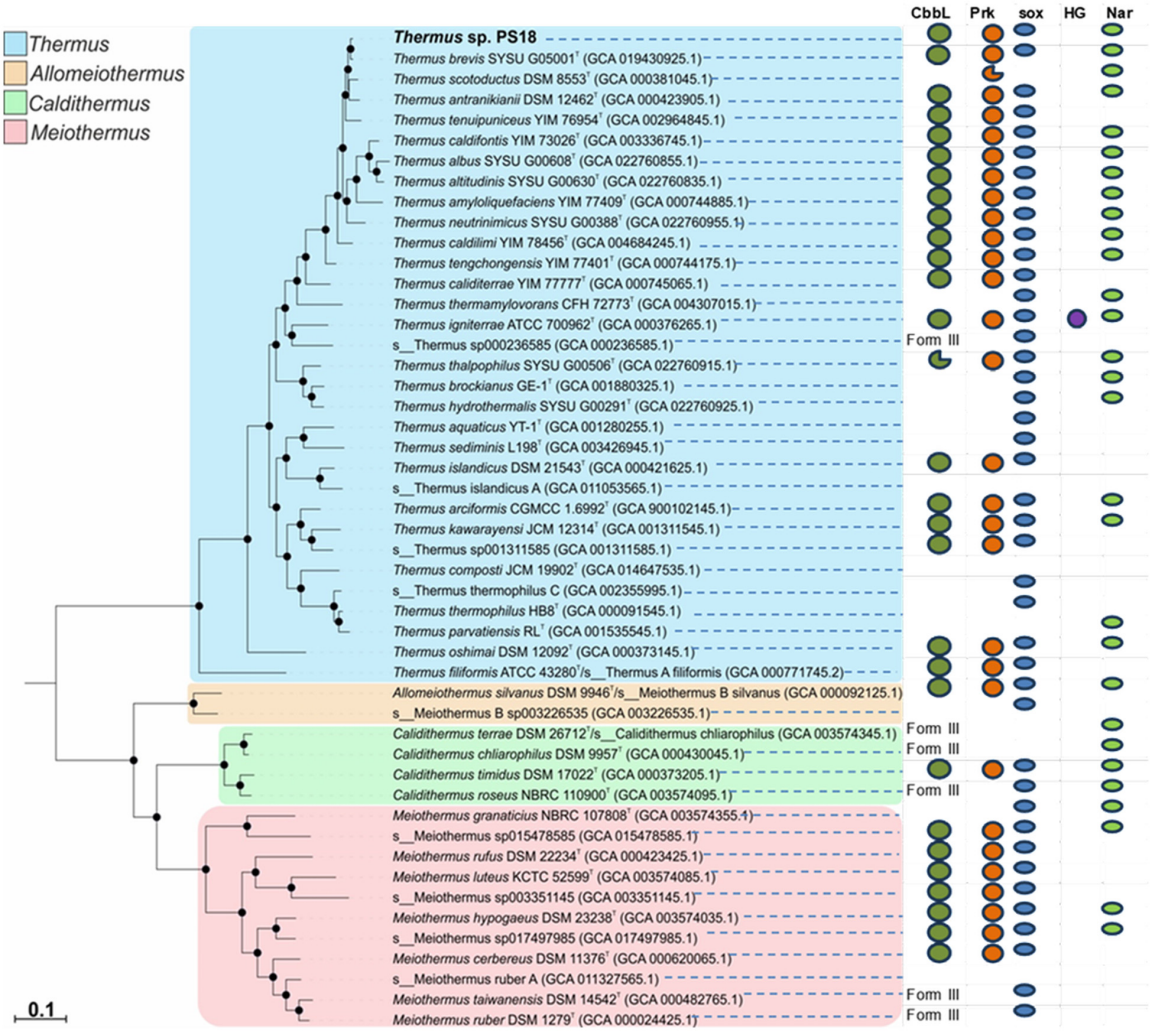
bac120 core proteins-based phylogenetic tree of type strains of *Thermaceae* family species, showing the position of isolate PS18. The outgroup was *Deinococcus radiodurans* R1T (not shown); black circles at nodes indicate support values of 95% or higher. Badges to the right of the tree illustrate distribution of genomic determinants of lithoautotrophic metabolism and nitrate reduction. CbbL, ribulose bisphosphate carboxylase large chain (form I, if not otherwise indicated); Prk, phosphoribulokinase; sox, sox gene cluster; HG, nickel-dependent uptake hydrogenase; Nar, nitrate reductase operon. The flawed circles denote truncated gene sequences.

Conserved protein-based phylogenetic analysis revealed position of *Thermus* sp. PS18 within *Thermaceae* family (Figure 4): the novel isolate belonged to *Thermus brevis*. The ANI value with the type strain of *T. brevis* was 98.0%, and the ANI values with type strains of other closest species were lower (95.4% with *T. antranikianii*, 94.8% with *T. scotoductus*, 94.0% with *T. tenuipuniceus*, 93.9% with *T. caldilimi*, and 90.2% with *T. tengchongensis*). The dDDH value (GGDC v.3.0, recommended formula 2) with the *T. brevis* type strain was 80.9%.

### Genomic determinants of autotrophic CO_2_ fixation by *T. brevis* PS18 and verification of their role by proteomic analysis

**Figure 5:**
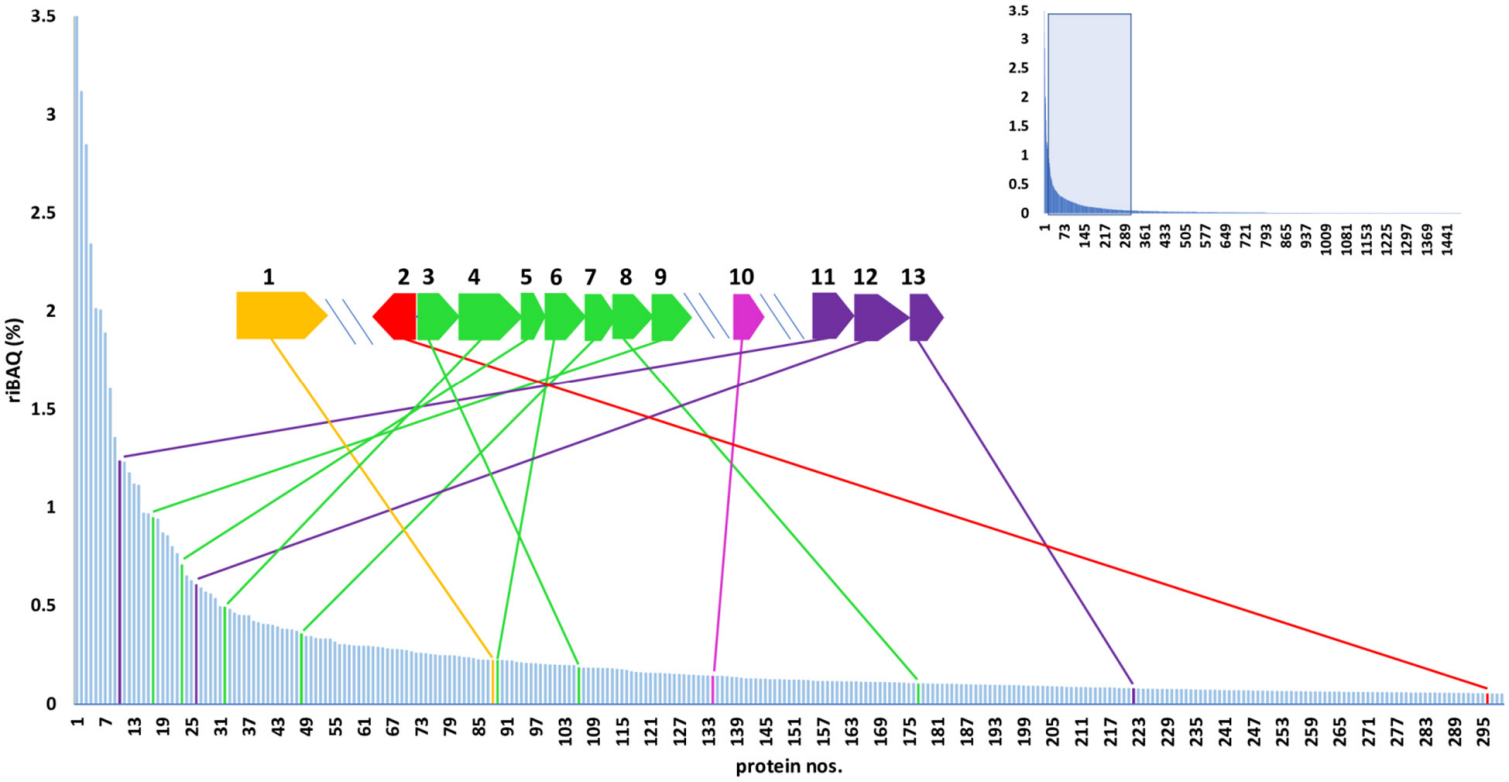
Overview of genomic and proteomic analysis results for *T. brevis* PS18 CBB cycle determinants. The proteins of aerobically autotrophically grown cells are sorted according to their relative abundances (riBAQ values, shown by Y axis), and 300 most abundant proteins are shown. The data for the 1511 proteins are outlined in the inset. The proteins encoded by the numbered genes are: 1, transketolase KQ693_12605; 2, transcriptional regulator of LysR family KQ693_12565; 3, class II fructose-bisphosphatase KQ693_12560; 4, ribulose-bisphosphate carboxylase large chain KQ693_12555; 5, ribulose bisphosphate carboxylase small subunit KQ693_12550; 6, red-type RuBisCo activase CbbX KQ693_12545; 7, phosphoribulokinase KQ693_12540; 8, ribulose-phosphate 3-epimerase KQ693_12535; 9, class II fructose-1,6-bisphosphate aldolase KQ693_12530; 10, ribose 5-phosphate isomerase KQ693_11515; 11, type I glyceraldehyde-3-phosphate dehydrogenase KQ693_09840; 12, phosphoglycerate kinase KQ693_09835; 13, triose-phosphate isomerase KQ693_09830. In case of isoforms of fructose-1,6-bisphosphate aldolase and fructose-1,6-bisphosphatase (more information in Supplementary Table 1), genomic and proteomic data are presented for isoforms with higher representation in the proteome.

Genomic analysis showed that the genome of *T. brevis* PS 18 contains all the determinants of the CBB cycle combined in four genomic loci (Figure 5, Supplementary Table 1). The genome also contains all the necessary genes for the reversible oxidative tricarboxylic acid cycle (roTCA cycle, Mall et al., 2018) and for the serine variant of the reductive glycine pathway (Sánchez-Andrea et al., 2020), see Supplementary Tables 2, 3. Based on the genome analysis and taking into account the currently recognized limitations imposed by high temperatures on the CBB cycle (Berg, 2011; Rasul et al., 2024), we could not rule out functioning of any of the three autotrophic pathways or their combined operation in our thermophilic isolate. Basis for more sound conclusions was to be provided by proteomic experiments.

**Figure 6:**
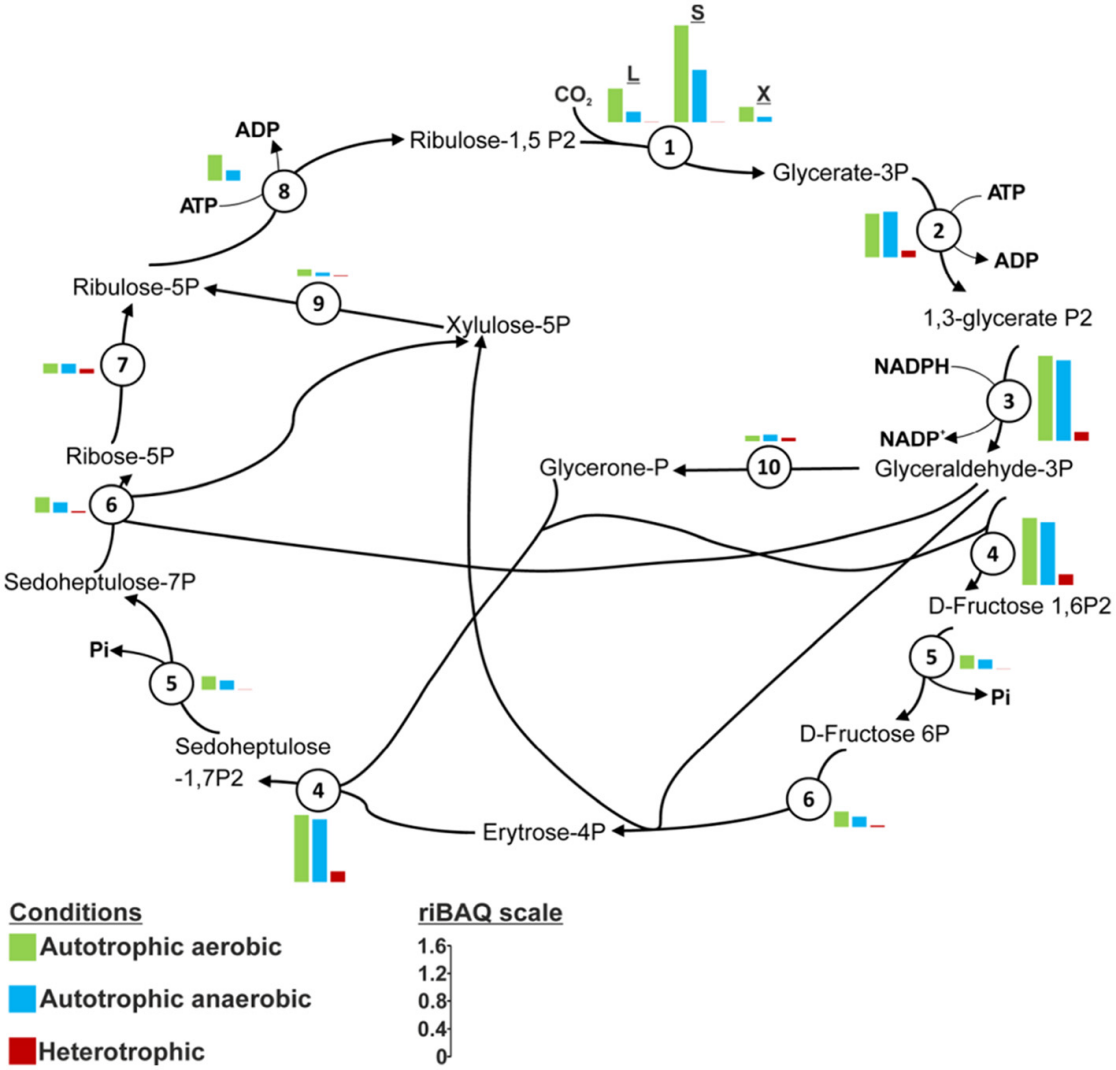
CBB cycle in *T. brevis* PS18 according to the determinants found in the genome and the expression of these determinants as dependent on cultivation conditions and revealed by proteomic analysis. 1, ribulose-bisphosphate carboxylase large subunit KQ693_12555; ribulose bisphosphate carboxylase small subunit KQ693_12550; 2, phosphoglycerate kinase KQ693_09835; 3, glyceraldehyde 3-phosphate dehydrogenase (phosphorylating) KQ693_09840; 4, class II fructose-1,6-bisphosphate aldolase KQ693_12530; 5, class II fructose-bisphosphatase KQ693_12560; 6, transketolase KQ693_12605; 7, ribose-5-phosphate isomerase KQ693_11515; 8, phosphoribulokinase KQ693_12540; 9, ribulose-phosphate 3-epimerase KQ693_12535; 10, triose-phosphate isomerase KQ693_09830. In case of isoforms of fructose-1,6-bisphosphate aldolase and fructose-1,6-bisphosphatase (more information in Supplementary Table 1) locus tags are indicated for isoforms with higher representation in the proteome.

The number of analyzed proteins in the proteomes obtained for different cultivation conditions varied from 1,462 to 1,511. The proteome analysis showed that each of them contained all the proteins of the CBB cycle and of the potential roTCA cycle. The CBB cycle enzymes were highly expressed in autotrophically grown cells (Figures 5, 6). In heterotrophically grown cells, the corresponding riBAQ values were significantly lower, the difference being quite dramatic for the key enzymes, RuBisCO and PRK (Figure 6). The riBAQ values for the enzymes of the potential roTCA cycle were much higher during heterotrophic growth, although expression of these enzymes was also observed during autotrophic growth (Supplementary Figure 1). In none of the cultivation variants did we record considerable expression of the enzymes of the potential glycine pathway serine variant (riBAQ values were no higher than 0.13%); the expression was lower under autotrophic conditions (Supplementary Figure 2). The obtained data indicate that *T. brevis* PS18, growing autotrophically at 65 °C, fixes CO__2__ both aerobically and anaerobically mainly or exclusively via the CBB cycle.

### Genomic determinants of lithotrophic metabolism in *T. brevis* PS18 and verification of their role by proteomic analysis

**Figure 7:**
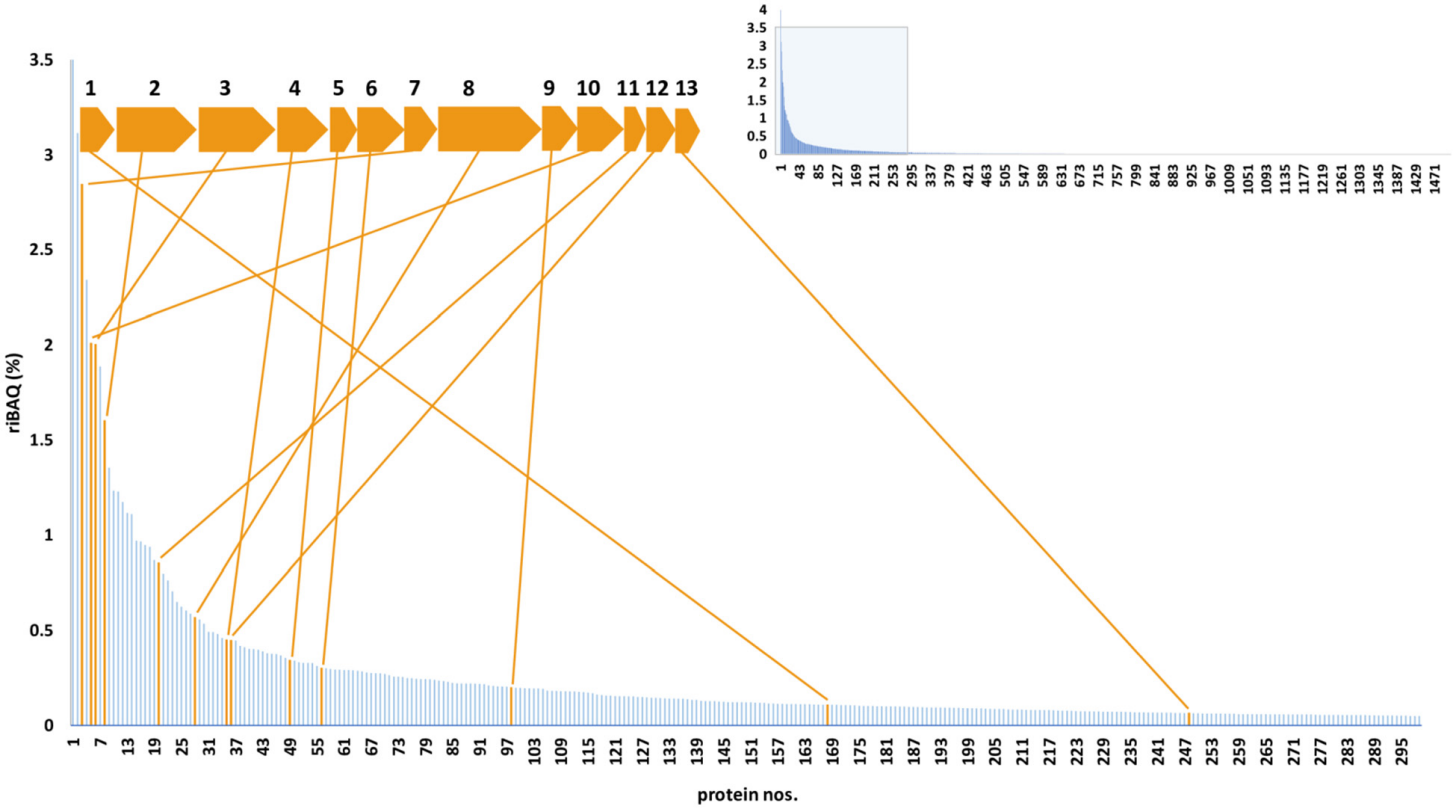
Overview of genomic and proteomic analysis results for *T. brevis* PS18 Sox system. The proteins of aerobically autotrophically grown cells are sorted according to their relative abundances in cell (riBAQ values, shown by Y axis), and the 300 most abundant proteins are shown. The data for the 1510 proteins are outlined in the inset. The proteins encoded by the numbered genes are: 1, thioredoxin family protein (KQ693_00460); 2, thiosulfate oxidation carrier protein SoxY (KQ693_00455); 3, thiosulfate oxidation carrier protein SoxZ (KQ693_00450); 4, sulfur oxidation c-type cytochrome SoxA (KQ693_00445); 5, sulfur oxidation c-type cytochrome SoxX (KQ693_00440); 6, thiosulfohydrolase SoxB (KQ693_00435); 7, sulfur oxidation c-type cytochrome SoxX (KQ693_00430); 8, sulfur oxidation c-type cytochrome SoxA (KQ693_00425); 9, rhodanese-like domain-containing protein (KQ693_00420); 10, translation initiation factor 2 (KQ693_00415); 11, NAD(P)/FAD-dependent oxidoreductase (KQ693_00410); 12, sulfite dehydrogenase SoxC (KQ693_00405); 13, cytochrome c SoxD (KQ693_00400).

As discussed above, strain PS18 was capable of sustainable lithoautotrophic growth by oxidation of thiosulfate to sulfate under aerobic conditions. Genomic analysis revealed that *T. brevis* PS18 genome contains the *sox* gene cluster known to be responsible for sulfur compound oxidation by diverse bacteria. In *T. brevis* PS18, the cluster comprised 13 genes (Figure 7, Supplementary Table 4), of which the genes encoding the four proteins SoxYZ, SoxXA, SoxB, and SoxCD, are thought to be necessary for thiosulfate oxidation to sulfate (Supplementary Figure 3; Friedrich et al., 2000, 2001; Murugapiran et al., 2013; Barrows and Van Dyke, 2023). The genes *sox*A and *sox*X were present in the cluster each in two copies, ca. 50%-identical in terms of the amino acid sequences of the encoded proteins.

Proteomic analysis showed that in *T. brevis* PS18 cells grown lithoautotrophically on thiosulfate the genes of the *sox* cluster were among highly expressed ones (Figure 7, Supplementary Figure 4).

*T. brevis* PS18 was isolated in aerobic conditions with CO as the substrate, but this growth mode was hardly reproducible in further work. Genome analysis of the strain has not revealed genes for CO dehydrogenases, either the [CuMo] or [NiFe] ones. It cannot be said that the [CuMo] CO dehydrogenase is completely out of character for *Thermus* spp., e.g., we found it to be encoded in the genome of *Thermus islandicus*. However, engagement of *T. islandicus* CO dehydrogenase as an additional blastp query did not help the cause of our isolate PS18. Notably, the sequence of the 16S rRNA gene in the genome was 100% identical to that determined earlier for CO-grown culture, dispelling doubts as to the purity of the initially obtained culture and favoring a hypothesis of a loss of a CO dehydrogenase-bearing plasmid during cultivation under unselective conditions.

In the *T. brevis* PS18 genome, no typical hydrogenases are encoded, either the [NiFe] or [FeFe] ones. There is a six-gene cluster (KQ693_10125–KQ693_10100) encoding an oxidoreductase that, based on its subunit composition and phylogeny, can formally be assigned to group 4f energy-converting [NiFe]-hydrogenases (Greening et al., 2016). However, its catalytic subunit KQ693_10110 lacks the nickel-binding CxxC motifs and, therefore, hydrogen is unlikely to be its substrate or product. Notably, we failed to find in the genome genes of proteins involved in hydrogenase nickel incorporation or hydrogenase maturation. The oxidoreductase KQ693_10125–KQ693_10100 is likely capable of transmembrane potential generation or consumption, but these processes are coupled with a redox reaction currently difficult to predict. These results of genome analysis are consistent with the lack of sustainable growth of the strain on hydrogen as electron donor.

### Genomic determinants of nitrate respiration in *T. brevis* PS18

In strain PS18 genome, the nitrogen compound respiration determinants (Simon and Klotz, 2013) are represented by genes for the membrane-bound nitrate reductase NarGHI, the cytochrome *cd1* nitrite reductase NirS, and NO-reductase NorBC (Supplementary Figure 5, Supplementary Table 5). The genomic organization of the determinants was generally similar to that described for *Thermus amyloliquefaciens* type strain YIM 77409^T^ and *T. tengchongensis* YIM 77401 (Zhou et al., 2016) and comprised several apparent transcription units. The largest of these is the *narCGHJIKK2* operon, which encodes the subunits of the NarGHI membrane-bound nitrate reductase, the NarJ chaperone, required for cofactor insertion into NarG, the NarC cytochrome *c*, and two NarK nitrate/nitrite transporters. Two bigenic operons, *dnrST* and *drpAB*, encoding regulatory proteins responsive to oxygen and nitrate, respectively (Sánchez-Costa et al., 2020), are located upstream and downstream of the *narCGHJIKT* operon. Not far upstream, there are genes encoding the NirS cytochrome *cd1*-type nitrite reductase and the NorBC nitric oxide reductase.

Nitrous oxide reductase gene, which is out of character for *Thermaceae* family, could not be found in strain PS18 genome. However, a gene annotated as nitrous oxide reductase accessory protein NosL did occur in the genome, as well as in the genomes of many *Thermaceae*.

*T. brevis* PS 18 grew at the expense of nitrate reduction to nitrite with formate (Figure 2B). In the genome, two formate dehydrogenases were encoded, one of which (KQ693_09550–09560) was a NAD-dependent enzyme, and the other, KQ693_08000–08010 (Supplementary Figure 6, Supplementary Table 6), was a membrane-associated enzyme similar in its predictable subunit composition to the well-studied *Escherichia coli* formate dehydrogenases FdoGHI and FdnGHI, involved in formate-oxidizing nitrate-reducing energy-converting complexes (Jormakka et al., 2003). The presumed KQ693_08000- and KQ693_080005-encoded subunits exhibit reasonably high identity levels (43–45% and 39–41%) with, respectively, the G and H subunits of Fdo and Fdn, whereas the KQ693_080010-encoded subunit (NrfD family protein) is not a homolog of subunit I (prokaryotic cytochrome *b561*), but is nevertheless its obvious functional analog in the complex, providing an electrogenic redox loop (Duarte et al., 2021). The presence in the operon of the gene of the accessory protein FdoD further increases the similarity of the *T. brevis* PS18 enzyme with the oxygen-tolerant Fdo (Benoit et al., 1998). Thus, we hypothesize that the growth of *T. brevis* PS18 at the expense of nitrate reduction to nitrite with formate is provided for by an energy-converting membrane-bound complex formed by the formate dehydrogenase KQ693_08000–08010 and the nitrate reductase NarGHI described above.

### Distribution of the key determinants of the CBB cycle and lithotrophy in *Thermaceae*

Our search for key CBB cycle determinants (RuBisCO and PRK) was conducted by blastp in NCBI nr database across *Deinococcota* phylum representatives. Only in the *Thermaceae* family were form I RuBisCO and PRK detected. Form II RuBisCO did not occur either in or beyond *Thermaceae*. In certain *Deinococcota* representatives beyond *Thermaceae*, form III RuBisCO was encountered, but these genomes lacked PRK genes. Form I and II RuBisCOs have long been known to be involved in the CBB cycle. The role of RuBisCO form III was for a long time thought to be restricted to the utilization of ribonucleotides and ribonucleosides via the pentose-bisphosphate pathway, but then a CBB cycle variant involving this enzyme form was discovered (Frolov et al., 2019). However, PRK remains indispensable from the currently recognized CBB cycle variants.

Within *Thermaceae* family, BLAST analysis revealed 18 species of *Thermus*, four species of *Meiothermus*, one species of *Calidithermus*, and one species of *Allomeiothermus* possessing CBB cycle key determinants in the genome (Figure 4). Some representatives of the family harbored form III RuBisCO genes but lacked PRK genes.

Most representatives of the *Thermaceae* family harbored *sox* genes, indicating their lithotrophic capacities (Figure 4). Complete sets of *sox* genes were revealed in 22 species of *Thermus*; 18 of those contained simultaneously CBB cycle determinants. Thus, thiosulfate oxidation is widespread in *Thermus*, and it is usually, but not necessarily, associated with the autotrophic potential.

### Autotrophic growth of *T. caldilimi* YIM 78456^T^

Among the type strains of *Thermus* species with CBB cycle and Sox enzymes encoded in genomes, we chose *T. caldilimi* YIM 78456^T^ to be tested for autotrophic capacity. The strain proved to be capable of sustainable autotrophic growth in aerobic conditions in mineral medium with thiosulfate as the energy source. The attained yield, about 7 × 10^7^ cells mL^−1^ (Supplementary Figure 7), was recorded over five transfers. During the growth, the pH of the medium decreased from pH 7.0 to pH 5.0, indirectly indicating the oxidation of thiosulfate to sulfate. Thus, proceeding from our genomic prediction, we demonstrated autotrophic growth of *T. caldilimi* YIM 78456^T^.

### Isolation, identification, and autotrophic growth of *Thermus* strains Uz8 and Uz79

Samples collected from a stream formed by the discharge of artesian groundwater in the Navoi region, Uzbekistan (see section Materials and methods), were used as inocula for enrichment cultures targeting autotrophic bacteria, resulting in the isolation of two strains, Uz8 and Uz79. Both strains were obtained from aerobic enrichment cultures established in media supplemented with reduced sulfur compounds and isolated by serial 10-fold dilution. Sodium thiosulfate (10 mM) was used for the enrichment and isolation of strain Uz8, whereas elemental sulfur (5 g L^−1^) was used for strain Uz79. Both isolates were represented by non-motile rod-shaped cells measuring 0.8–1 × 0.5 μm (Uz8, Supplementary Figure 8) and 1–2 × 0.4 μm (Uz79, Supplementary Figure 9) and exhibited facultatively autotrophic lifestyle, being also capable of growth on complex organic substrates such as yeast extract or peptone. The strains showed stable autotrophic growth under aerobic conditions with thiosulfate or elemental sulfur as electron donor; no growth was observed with molecular hydrogen as the energy source.

Based on 16S rRNA gene sequence analysis, strain Uz8 was assigned to the species *Thermus oshimai* (Williams et al., 1996), showing 98.7% and 99.0% sequence similarity to *T. oshimai* strains SPS-17^T^ and JL-2, respectively. Previously, metagenomic sequencing of the DNA isolated from the same sample was performed, and MAG *Thermus* sp. U4-19 was assembled and submitted to GenBank (GCA_037481675.1, Slobodkina et al., 2025). This MAG shows 87.8 and 86.1% dDDH levels with *Thermus oshimai* strains DSM 12092^T^ and JL-2 and harbors a *sox* gene cluster and all the necessary genes of the CBB cycle. Thus, the U4-19 MAG and the isolate Uz8 likely represent the same natural population of *T. oshimai*, which may be among primary producers in the habitat, although the abundance of U4-19 phylotype in the microbial community was estimated to be as low as 1.17%.

The other isolate, strain Uz79, was affiliated with the species *Thermus scotoductus*, exhibiting 16S rRNA gene sequence similarities of 98.4% to *T. scotoductus* SE-1^T^ and 99.3% to *T. scotoductus* SA-01. The draft genome of strain Uz79 was assembled into 95 scaffolds with total length of 2,374,843 bp and GC content of 65.1%. According to the CheckM estimation, completeness and contamination of the assembly were 95.2% and 2.9%, respectively. The annotation revealed 2,490 protein-coding genes and 52 RNA genes including 46 tRNA genes, 3 rRNA genes (5S, 16S, 23S) and 3 ncRNA. The dDDH levels with the type strains of closest *Thermus* species were 60–69%, but this value was 82.0% with strain *T. scotoductus* SA-01 (Kristjansson et al., 1994), whose affiliation with the *T. scotoductus* species is confirmed by the current GTDB R226 phylotaxonomy. The *T. scotoductus* Uz79 genome contained genes of the CBB cycle and genes of thiosulfate oxidation (*sox* cluster), which was in accordance with the strain's cultural properties.

### On the origin and evolution of autotrophy in *Thermaceae*

**Figure 8:**
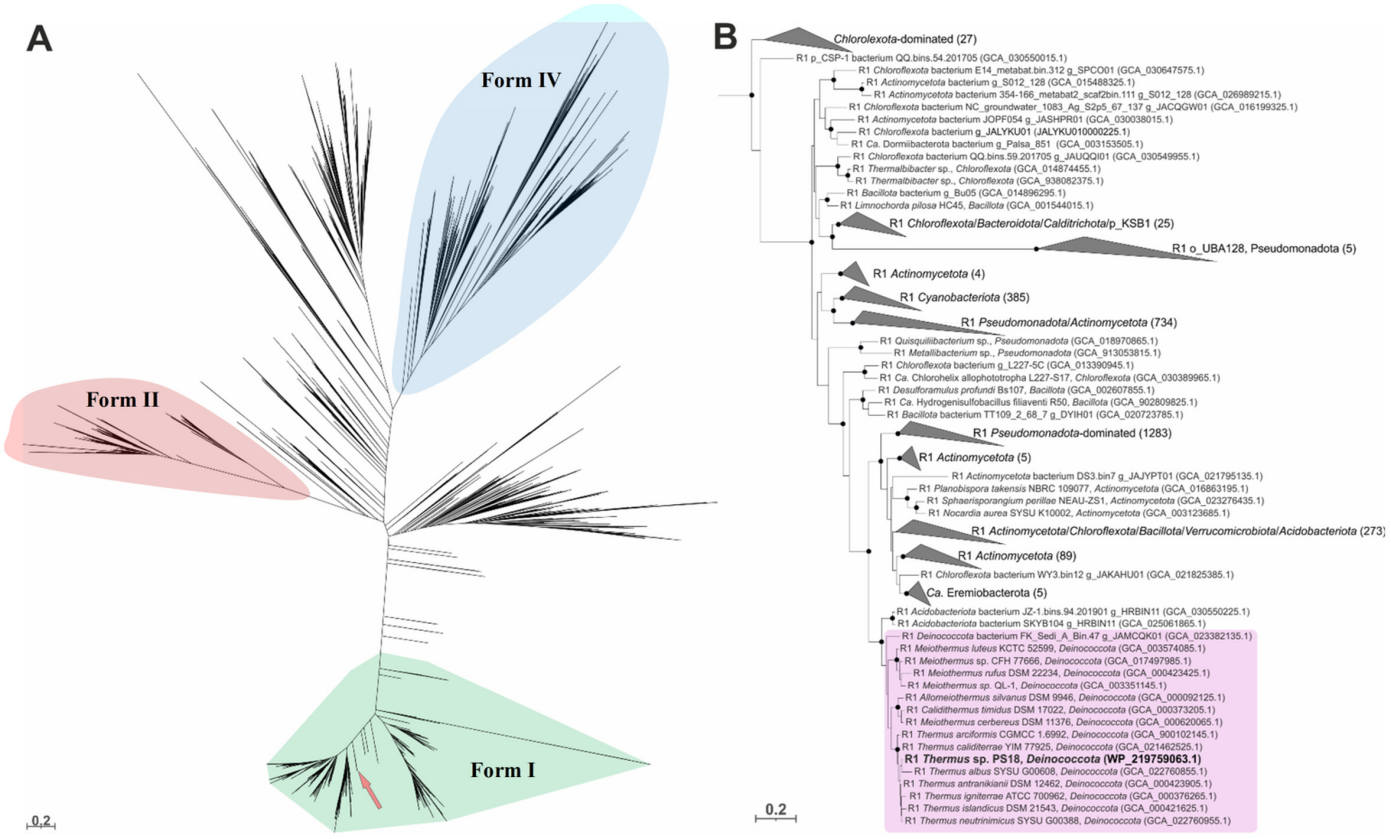
Maximum likelihood phylogenetic tree for 5487 RuBisCO large subunit amino acid sequences: **(A)** Full tree. Branches that are not within colored polygons belong to various subtypes of RuBisCO form III, which tend to be scattered over present-day RuBisCO trees (Liu et al., 2023). The pink arrow shows the position of *Thermaceae* representatives. **(B)** Subtree of the full tree, comprising form I RuBisCOs. RuBisCOs of the *Thermaceae* family representatives are within the purple box. *Thermus* sp. PS18 RuBisCO is in bold. Black circles at nodes indicate bootstrap support higher than 90% of 1,000 replicas.

Within the *Deinococcota* phylum, *Thermaceae* is the only family whose members harbor form I RuBisCO and PRK genes. These genes occur within a 7-gene presumable operon (hereafter, *cbb* gene cluster; genes 3 to 9 in Figure 5) conserved in *Thermaceae* spp. with minor variations. In RubisCO phylogenetic tree, the enzymes of *Thermaceae* representatives form a monophyletic subclade (Figure 8), arising at a moderate evolutionary depth within the form I RuBisCO clade. Thus, the form I RuBisCO and PRK genes seem to have been acquired by the *Thermaceae* lineage common ancestor via HGT that occurred not long before the lifetime of the lineage last common ancestor.

As for the origin of the lithotrophic capacities of *Thermaceae* spp., our analysis showed that closest homologs of *Thermaceae soxC* and *soxD* occur outside of *Deinococcota* phylum (Supplementary Figure 10). Without in-depth analysis of *sox* genes phylogeny, this distribution pattern of homologs allows us to conclude that the mechanism of sulfur compound oxidation currently functioning in *Thermacease* spp. was acquired by their ancestor by HGT in the same period as the CBB cycle determinants.

**Figure 9:**
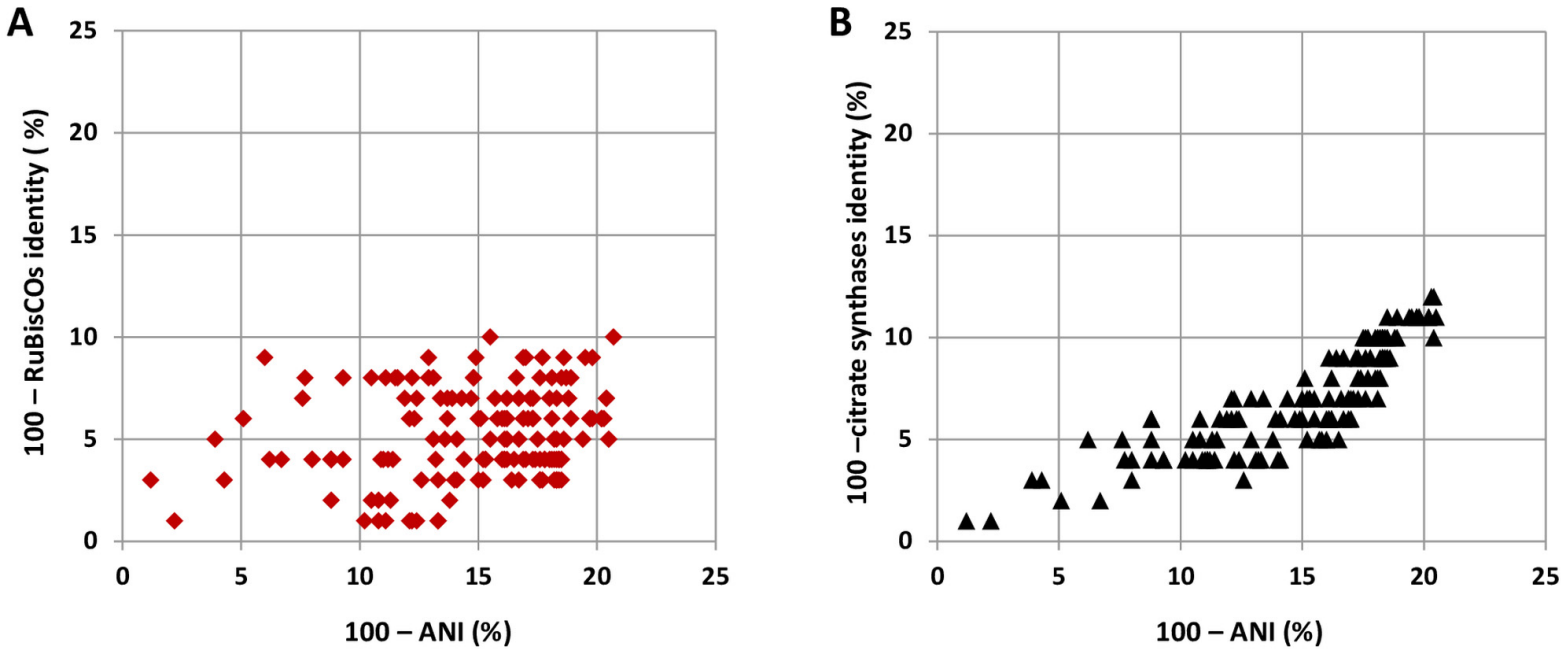
Bioinformatic evaluation of horizontal mobility of **(A)** RuBisCO and **(B)** citrate synthase genes within the genus *Thermus*. Phylogenetic distances within pairs of orthologs, evaluated as percentage of distinctions between their amino acid sequences (100—identity, %), are plotted against phylogenetic distances between the host strains, evaluated as percentage of distinctions between nucleotide sequences of host genomes (100—ANI, %). All RuBisCO-harboring type strains of *Thermus* spp. and a few RuBisCO-harboring non-type strains of *Thermus* spp. were compared with each other in terms of their RuBisCOs and citrate synthases; plotted are data for each pair.

In the course of the evolution that followed these HGT events, there was no further input of RuBisCO genes from the outside of the *Thermaceae* phylogenetic lineage, and at a first glance the RuBisCO inheritance pattern within the family may be taken for vertical (Figure 8B, Supplementary Figure 11). Closer examination reveals notable deviations: these are, e.g., the RuBisCOs of *Allomeiothermus silvanus* and *Calidithermus timidus*, which in phylogeneic trees fall within the radiation of *Meiothermus* spp. RuBisCOs with strong bootstrap support (Figure 8B, Supplementary Figure 11). As for the RuBisCO tree topology within the genus *Thermus*, the compositions of the clades formed by *Thermus* spp. type strains were not quite the same in the RuBisCO tree and the core proteins tree (cf. Supplementary Figure 11, Figure 4). It may be said that these inconsistencies can be explained by the insufficient resolving power of the RuBisCO treeing method (low bootstrap values in Supplementary Figure 11 have to be admitted). However, high lateral mobility of the RuBisCO gene within the *Thermaceae* clade can be corroborated by demonstrating weakness of the correlation between phylogenetic distances between *Thermus* spp. strains and those between their RuBisCOs. Bright examples are, e.g., the *T. scotoductus* Uz79–*T. islandicus* DSM 21543^T^ pair (99.2% identical RuBisCOs at only 82% AAI of total proteomes: closely related RuBisCOs in remote species) and the *T. albus–T. altitudinis* type strains pair (91.8% identical RuBisCOs at 94.2% AAI of total proteomes: remote RuBisCOs in close species). Figure 9 illustrates high lateral mobility of RuBisCO genes in the genus *Thermus* by showing weakness of the discussed correlation on a broader scale (Figure 9A). The correlation was much stronger for the reference protein that we chose, citrate synthase (Figure 9B). We believe that the difference between the inheritance patterns of the two enzymes should be explained by presumably facultative role of the CBB cycle in *Thermaceae*, provoking evolution of its key determinants to involve repeated gene losses and reacquisitions from the pangenome. While replacement of citrate synthase via HGT must be a randomly occurring and evolutionarily neutral event, the losses and reacquisitions of RuBisCO may be supported by natural selection, and this explains the notably higher rates of these events, evident from Figure 9.

The survival strategy involving gene loss and reacquisition under variable environmental conditions may be promoted by the natural competence inherent in *Thermus* spp. (Koyama et al., 1986; Gounder et al., 2011), and by their reported transconjugation capacity (Blesa et al., 2017, 2020), providing for the putative ease of the gene reacquisition.

This strategy can best be traced at intraspecies level. Within the *Thermaceae* family, two species are represented by numerous strains with sequenced genomes. These are *T. thermophilus* (41 genomes in NCBI GenBank as of July 2025) and *T. scotoductus* (46 genomes). In the former species, none of the strains harbors RuBisCO or PRK gene. In *T. scotoductus*, the occurrence pattern of CBB cycle determinants is sophisticated. We believe that this pattern may shed light on the (micro)evolutionary mechanisms of the CBB cycle inheritance, at least in *Thermaceae*; therefore, we would like to consider the inheritance patterns of CBB cycle determinants within the *T. scotoductus* species in more detail, the more so that our isolates Uz79 belongs to this species, while our isolate *T. brevis* PS18 is from a species closely related to it.

The type strain of the species, *T. scotoductus* DSM 8553^T^, was isolated from hot tap water in Iceland (Kristjansson et al., 1994), 39 strains were isolated from household water heaters all over United States (Wilpiszeski et al., 2019), and six are isolates from natural hot springs.

**Figure 10:**
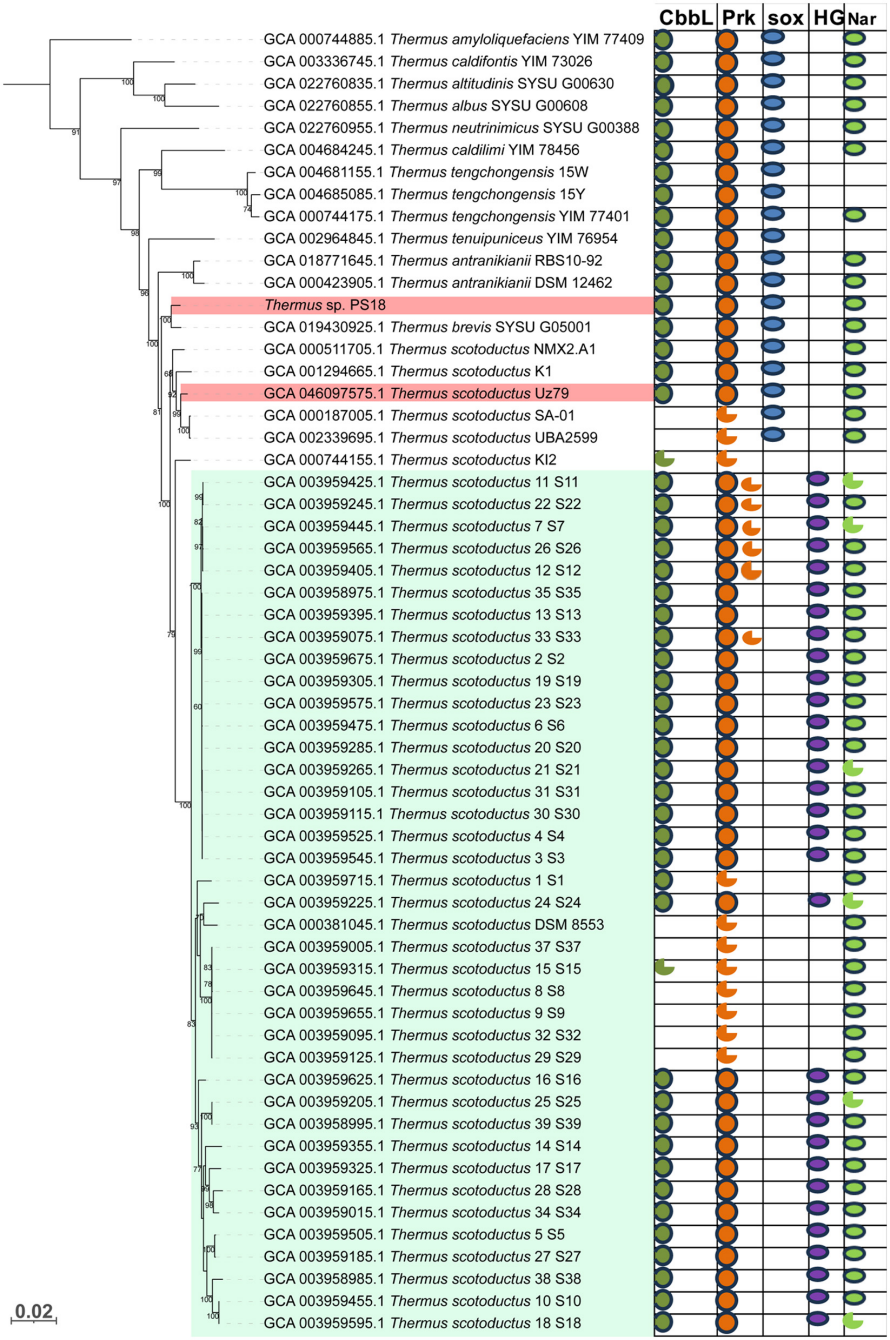
Distribution of genomic determinants of lithoautotrophic metabolism and nitrate reduction among closest relatives of strain PS18 within the genus *Thermus*. Phylogenetic tree was constructed based on bac120 core proteins. CbbL, form I ribulose bisphosphate carboxylase large chain; Prk, phosphoribulokinase; sox, sox gene cluster; HG, nickel-dependent uptake hydrogenase; Nar, nitrate reductase operon. The light green background highlights the *T. scotoductus* strains isolated from household water heaters. The pink background highlights our isolates. The flawed circles denote truncated gene sequences.

Among the 46 *T. scotoductus* strains, 11 (including the type strain *T. scotoductus* DSM 8553^T^ and eight water heater isolates) lacked RubisCO genes, as well as genes of lithotrophic metabolism (*sox* genes; Figure 10). Notably, they contained remnants of the *cbb* gene cluster, including a truncated PRK gene (flawed circles in Figure 10) and genes of ribulose-phosphate 3-epimerase and class II fructose-1,6-bisphosphate aldolase, adjoining it downstream. The rest 35 strains harbored *cbb* gene clusters with RuBisCO genes that all belonged to the *Thermaceae* phylogenetic lineage but represented three sublineages. Two of these sublineages were only 0.6–1.7% different from closest RuBisCOs belonging to type strains of other *Thermus* species (hereafter, *T. albus*-like sublineage and *T. islandicus*-like sublineage; this proximity can also so be seen in the tree in Supplementary Figure 11), The third *T. scotoductus* RuBisCO sublineage did not have so closely related alien RuBisCOs (hereafter, incognito sublineage). None of these RubisCOs seem to have been inherited from the *T. scotoductus* common ancestor, since they are not close to RuBisCOs of the species closest to *T. scotoductus*, such as *T. brevis* and *T. antranikianii*.

It is hardly possible to exactly trace the series of the events that led to the observed occurrence pattern of these RuBisCO variants, but, in addition to gene replacements, they most likely included RuBisCO gene losses and reacquisitions (see argumentation below).

The *T. scotoductus* strains with RuBisCO of the “*T. albus*-like” sublineage were natural hot-spring isolates, and they harbored *sox* genes. Among strains with RuBisCOs of the other two sublineages, all were water heater isolates and they lacked *sox* genes and harbored instead genes of a hydrogenase (Figure 10) belonging to group 2 cytosolic uptake [NiFe]-hydrogenases.

**Figure 11:**
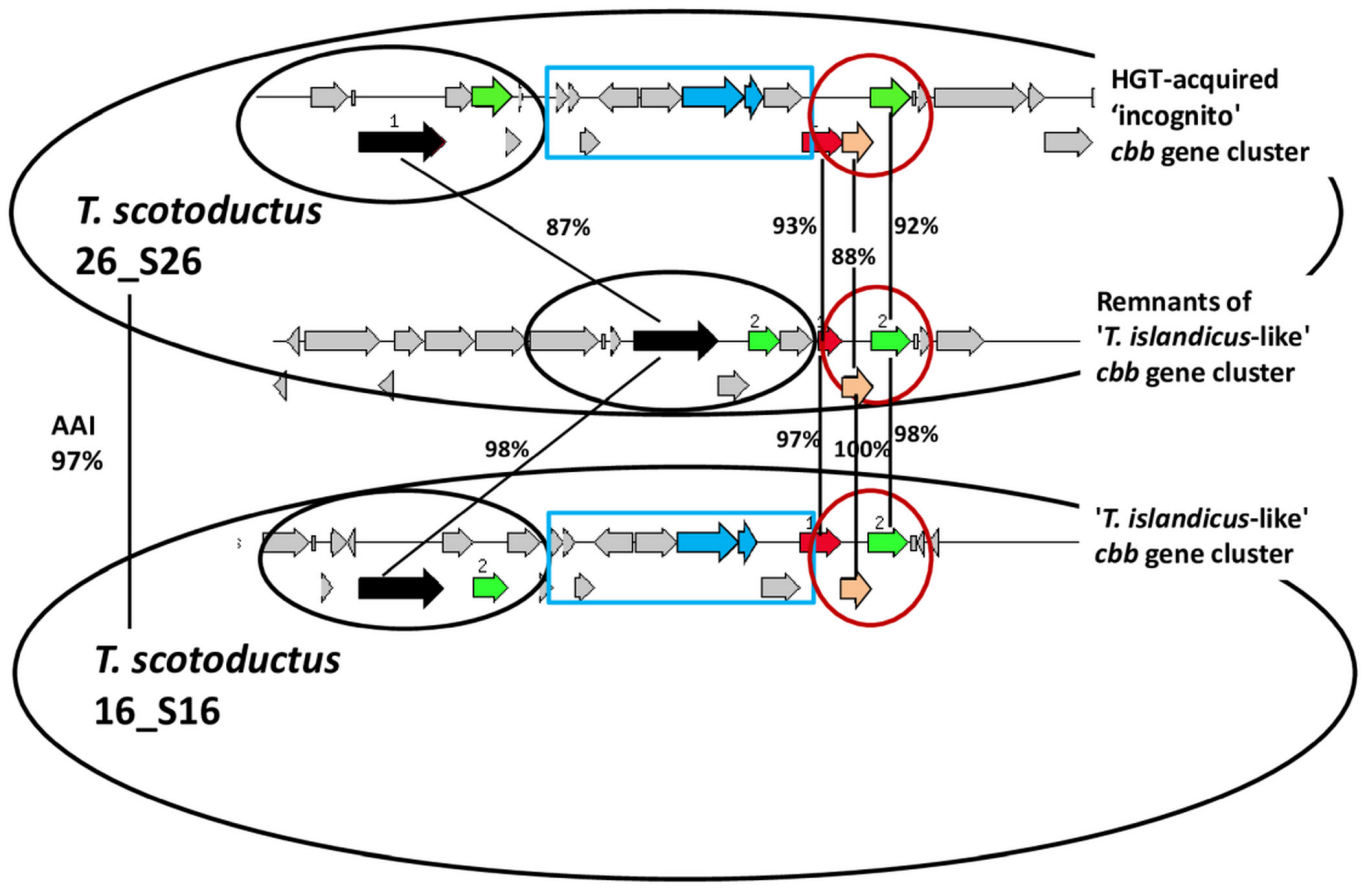
Organization of *cbb* gene clusters in two water heater isolates of *T. scotoductus*. The “*T. islandicus*-like” *cbb* gene cluster was evidently inherited vertically from the common ancestor; the “incognito” gene cluster must have been acquired by HGT. Circled are syntenic loci; amino acid sequence identity is shown for deduced products of some genes. In rectangle is the RuBisCO-containing locus that is lacking in the impaired “*T. islandicus*-like” gene cluster. Black arrows, transketolase genes; green arrows, genes of class II fructose-1,6-bisphosphate aldolase; blue arrows, genes of RuBisCO large and small subunits; red arrows, phosphoribulokinase genes; orange arrows, genes of ribulose-phosphate 3-epimerase.

Presumably, shortly before water heater colonization or at its initial stage, the common ancestor of the water heater *T. scotoductus* strains lost *sox* genes and the RuBisCO gene. Then, it acquired genes for an uptake [NiFe]-hydrogenase and reacquired the *cbb* gene cluster, The likely ecological cause for *sox* genes loss and hydrogenase genes acquisition is that the water fed into the heaters is normally poor in sulfur compounds, whereas hydrogen may be produced by various types of the equipment corrosion (Khoshnaw, 2024). As for the losses and (re)acquisitions of RuBisCO genes, their cause may be e.g., irregular treatment with corrosion inhibitors, yielding available organic substrates upon thermal decomposition (Khoshnaw, 2024). Notably, the reacquired *cbb* gene cluster was with RuBisCO of another, “*T. islandicus*-like,” phylogenetic sublineage. One branch of further progeny has retained this “*T. islandicus*-like” *cbb* gene cluster up to the present time; in the other branch, it was replaced by an “incognito” *cbb* gene cluster. Remarkably, this replacement cannot be easily interpreted as replacement by homologous recombination, since, in some of these strains, remnants of the old, “*T. islandicus*-like” *cbb* gene cluster have been retained, including truncated PRK gene (Figures 10, 11). The genomes of these six strains (22_S22, 11_S11, 7_S7, 12_S12, 26_S26, and 33_S33) deserve special attention. Since remnants of the old *cbb* gene cluster are present in these genomes, the first idea is that the acquisition of the new one must have occurred by non-homologous recombination. The genome assemblies of the discussed strains comprised hundreds of contigs, but these were six genome assemblies of very closely related strains, and we thought that, by docking the contigs of the six strains, as well as by mapping them onto an available complete chromosome of a related outgroup *Thermus* strain, we would be able to assemble a chimeric circled chromosome that would give an idea of the organization of the naturally occurring chromosome and of the site of the insertion of the newly acquired *cbb* gene cluster. These attempts however failed. Surprisingly, we found the new *cbb* gene cluster and the remnants of the old one to occur in syntenic genomic contexts comprising about 80 genes (Supplementary Table 7). These 80 genes were present in the genome assemblies in two homologous (ca. 90%-identical) copies. No other regions of this kind could be found in the assemblies, so the observed phenomenon could not result from culture impurity. On the edges of the syntenic regions, the copies were competitors for the sites of docking with the rest of the chromosome.

We could not find in these enigmatic regions genes that would indicate their plasmid nature. Therefore, our hypothesis is heterozygous diploidy, i.e., sustainable presence in the cell of non-identical chromosome copies (or non-identical sets of identical copies). The bulks of the non-identical copies are identical (indistinguishable in the course of sequencing and assembly), but the discussed 80-gene fragments are not. We think that the new chromosome originated from homologous recombination of one of the original strands with an alien 80-gene DNA fragment. After replication, this gave rise to heterozygous state, which has been maintained over generations due to unknown reasons and mechanisms. Not that the reasons and mechanisms cannot be hypothesized. Natural selection may be the reason and the mechanism: the new fragment provides autotrophic machinery but it is probably impaired in some vital property. At the moment of the acquisition of the new fragment, the old one was most likely already impaired in the autotrophic determinants (i.e., loss of the property preceded its (re)acquisition). That is, the heterozygosity maintenance mechanism may in this case be in principle similar to that implemented in artificially engineered heterozygous bacterial constructs (Li, 2019; Wang et al., 2023). Polyploidy is well known for *Deinococcota* representatives, and it has been documented for *T. thermophilus* (Ohtani et al., 2010; Li, 2019); However, heterozygous state has only been reported for *T. thermophilus* engineered constructs (Li, 2019). Our examination of the genomes of *T. scotoductus* isolates allow us to assume that this may be a natural phenomenon, albeit perhaps transient on the evolutionary time scale. The reason for the lack of other detectable differences in the sister chromosomes of the discussed *T. scotoductus* strains, as well as the failure of the host organisms to return to homozygous state by recombination between sister chromosomes, may be the short amount of time that has elapsed after the establishment of heterozygosity, which took place in the course of accelerated evolution in a new anthropogenic ecological niche (water heaters).

## Conclusion

Thus, by the isolation of autotrophically growing strains of the genus *Thermus*, analysis of the available genomes of representatives of this genus, and comparative proteomic analysis of *T. brevis* PS18, we show that representatives of the genus *Thermus* are capable of autotrophy due to the operation of the CBB cycle and thus can be among primary producers in thermal habitats under conditions of organic carbon deficiency.

*Thermus* spp. have been among model organisms for studying life at high temperatures, and many aspects of biology of these bacteria have been thoroughly studied. It is a puzzling fact that their autotrophic capacities could have escaped researchers' attention for more than half a century. Anyway, this period of researchers' unawareness of this important capacity of *Thermus* bacteria has come to its end. Bioengineering tools for *Thermus* species are well-developed, and the results presented in this paper can serve as a basis for engineering experiments with CBB cycle enzymes operating at high temperatures.

Our bioinformatic insight into the evolution of the autotrophic capacity in the family *Thermaceae* revealed remarkable lateral mobility of autotrophy key determinants in this family, which we explain in terms of our hypothesis of inheritance of facultative characters by gene loss and reacquisition from the pangenome. We also argue that in *Thermaceae* the (re)acquisition mechanism may involve heterozygous stage sustainable over generations.

## Data Availability

The genome sequences are available in NCBI GenBank under accession numbers CP102606 (*T. brevis* PS18 chromosome), CP102607 (*T. brevis* PS18 plasmid), and JBJUSP010000000 (*T. scotoductus* Uz79 whole genome shotgun sequence). Partial sequences of 16S rRNA genes of *T. brevis* PS18, *T. oshimai* Uz8, and *T. scotoductus* Uz79 are available in NCBI GenBank under accession numbers PX879835, PX463433, and PX879155. The mass spectrometry proteomics data have been deposited to the ProteomeXchange Consortium (Deutsch et al., 2023) via the PRIDE partner repository (Perez-Riverol et al., 2025) with the dataset identifier PXD070097.
