# Supplementary material for: Autotrophic growth of Thermus sp. PS18 and its genomic determinants shed light on the autotrophic lifestyle and its evolution in the Thermaceae family
Source: Front Microbiol. 2026 Mar 12;17:1769897. doi: 10.3389/fmicb.2026.1769897 (PMC13019369; doi:10.3389/fmicb.2026.1769897)
Supplement: Supplementary file 1 [file Table_1.docx]

**Supplementary Table 1.** CBB cycle enzymes of *T. brevis* PS18

| **Enzyme*** | **GenBank**  **Locus tag** | **AutAer**  **rank**** | **AutAna**  **rank**** | **Hetero**  **rank**** |
| --- | --- | --- | --- | --- |
| Ribulose-bisphosphate carboxylase large chain [EC:[4.1.1.39](https://www.genome.jp/entry/4.1.1.39)] | KQ693_12555 | 31 | 114 | 1239 |
| Ribulose bisphosphate carboxylase small subunit [EC:[4.1.1.39](https://www.genome.jp/entry/4.1.1.39)] | KQ693_12550 | 22 | 17 | 1362 |
| Phosphoglycerate kinase [EC:2.7.2.3] | KQ693_09835 | 25 | 24 | 167 |
| Type I glyceraldehyde-3-phosphate dehydrogenase [EC:[1.2.1.12](https://www.genome.jp/entry/1.2.1.12)] | KQ693_09840 | 10 | 8 | 122 |
| Class II fructose-1,6-bisphosphate aldolase [EC:4.1.2.13] | KQ693_12530 | 16 | 14 | 110 |
| Class II fructose-bisphosphatase [EC:3.1.3.11] | KQ693_12560 | 105 | 141 | 1333 |
| Transketolase [EC:[2.2.1.1](https://www.genome.jp/entry/2.2.1.1)] | KQ693_12605 | 87 | 123 | 536 |
| Ribose-5-phosphate isomerase [EC:5.3.1.6] | KQ693_11515 | 133 | 139 | 218 |
| Phosphoribulokinase [EC:2.7.1.19] | KQ693_12540 | 47 | 124 | - |
| Ribulose-phosphate 3-epimerase [EC:5.1.3.1] | KQ693_12535 | 177 | 284 | 851 |
| Triose-phosphate isomerase [EC:5.3.1.1] | KQ693_09830 | 221 | 193 | 296 |
| Transcriptional regulator, LysR family | KQ693_12565 | 295 | 409 | 1365 |
| CbbX protein | KQ693_12545 | 88 | 229 | - |
| class II fructose-bisphosphatase [EC: 3.1.3.11] | KQ693_11940 | 408 | 505 | 584 |
| Fructose-bisphosphate aldolase/bisphosphatase [3.1.3.11] | KQ693_08830 | - | - | - |
| Fuculose-1-phosphate aldolase [4.1.2.17] | KQ693_04600 | 763 | 620 | 522 |
| Class II fructose-bisphosphate aldolase [EC 4.1.2.13] | KQ693_12595 | 543 | 535 | 1422 |
| Deoxyribose-phosphate aldolase [EC 4.1.2.4] | KQ693_10845 | 758 | 1020 | 322 |

*Enzyme annotations originate from manual curation of RAST and GenBank (GCA_026427635.1) annotations.

**Ranks in the list of proteome proteins arranged according to their relative molar abundances (riBAQ values) in descending order; dash means 'not represented'. AutAer, AutAna, Hetero – the three variants of cell growth: autotrophic aerobic, autotrophic anaerobic, heterotrophic (see main text).

Isoenzymes with lower presentation in proteome are shadowed gray
