# Supplementary material for: Autotrophic growth of Thermus sp. PS18 and its genomic determinants shed light on the autotrophic lifestyle and its evolution in the Thermaceae family
Source: Front Microbiol. 2026 Mar 12;17:1769897. doi: 10.3389/fmicb.2026.1769897 (PMC13019369; doi:10.3389/fmicb.2026.1769897)
Supplement: Supplementary file 2 [file Table_2.docx]

**Supplementary Table 2.** Enzymes of the potential roTCA cycle in *T. brevis* PS18

| **Enzyme*** | **GenBank**  **locus tag** | **AutAer**  **rank**** | **AutAna**  **rank**** | **Hetero**  **rank**** |
| --- | --- | --- | --- | --- |
| Citrate synthase (si) (EC 2.3.3.1) | KQ693_06075 | 241 | 226 | 61 |
| Pyruvate:ferredoxin (flavodoxin) oxidoreductase (EC 1.2.7.1) | KQ693_09635 | 920 | 1354 | 1424 |
| Phosphoenolpyruvate synthase (EC 2.7.9.2) | KQ693_00705 | 845 | 1013 | 1255 |
| Phosphoenolpyruvate carboxylase (EC 4.1.1.31) | KQ693_09960 | 939 | 977 | 1062 |
| Malate dehydrogenase (EC 1.1.1.37) | KQ693_06430 | 459 | 279 | 194 |
| Fumarate hydratase class II (EC 4.2.1.2) | KQ693_07060 | 597 | 430 | 222 |
| Succinate dehydrogenase (EC 1.3.5.1), iron-sulfur subunit | KQ693_12015 | 510 | 357 | 572 |
| Succinate dehydrogenase (EC 1.3.5.1), flavoprotein subunit | KQ693_12020 | 903 | 506 | 186 |
| Succinyl-CoA ligase [ADP-forming] (EC 6.2.1.5), alpha subunit | KQ693_06960 | 395 | 519 | 71 |
| Succinyl-CoA ligase [ADP-forming] (EC 6.2.1.5), beta subunit | KQ693_06965 | 317 | 1424 | 48 |
| 2-Oxoglutarate:ferredoxin oxidoreductase (EC 1.2.7.3), beta subunit | KQ693_02940 | 1030 | 1434 | 734 |
| 2-Oxoglutarate:ferredoxin oxidoreductase (EC 1.2.7.3), alpha and gamma subunits | KQ693_02935 | 689 | 1164 | 173 |
| Isocitrate dehydrogenase [NADP] (EC 1.1.1.42) | KQ693_11820 | 496 | 487 | 97 |
| Aconitate hydratase (EC 4.2.1.3) | KQ693_07290 | 596 | 653 | 248 |

*Enzyme annotations originate from manual curation of RAST and GenBank (GCA_026427635.1) annotations.

**Ranks in the list of proteome proteins arranged according to their relative molar abundances (riBAQ values) in descending order; dash means 'not represented'. AutAer, AutAna, Hetero – the three variants of cell growth: autotrophic aerobic, autotrophic anaerobic, heterotrophic (see main text).
