# Supplementary material for: Autotrophic growth of Thermus sp. PS18 and its genomic determinants shed light on the autotrophic lifestyle and its evolution in the Thermaceae family
Source: Front Microbiol. 2026 Mar 12;17:1769897. doi: 10.3389/fmicb.2026.1769897 (PMC13019369; doi:10.3389/fmicb.2026.1769897)
Supplement: Supplementary file 3 [file Table_3.docx]

**Supplementary Table 3.** Enzymes of the potential reductive glycine pathway serine variant in *T. brevis* PS18

| **Enzyme*** | **GenBank**  **locus tag** | **AutAer**  **rank**** | **AutAna**  **rank**** | **Hetero**  **rank**** |
| --- | --- | --- | --- | --- |
| NAD-dependent formate dehydrogenase alpha subunit | KQ693_09560 | 1259 | 1330 | 1498 |
| 2Fe-2S iron-sulfur cluster-binding protein | KQ693_09555 | 0 | 0 | 0 |
| NAD-dependent formate dehydrogenase, NuoF-like subunit | KQ693_09550 | 961 | 1332 | 1477 |
| formate--tetrahydrofolate ligase | KQ693_03985 | 806 | 914 | 721 |
| bifunctional 5,10-methylene-tetrahydrofolate dehydrogenase/5,10-methylene-tetrahydrofolate cyclohydrolase | KQ693_07895 | 879 | 959 | 371 |
| aminomethyl-transferring glycine dehydrogenase subunit GcvPB | KQ693_06350 | 655 | 773 | 337 |
| aminomethyl-transferring glycine dehydrogenase subunit GcvPA | KQ693_06345 | 678 | 769 | 222 |
| glycine cleavage system protein GcvH | KQ693_06340 | 633 | 429 | 264 |
| glycine cleavage system aminomethyltransferase GcvT | KQ693_06335 | 338 | 360 | 140 |
| serine hydroxymethyltransferase | KQ693_00830 | 209 | 273 | 130 |
| L-serine ammonia-lyase, subunit alpha | KQ693_09900 | 801 | 857 | 968 |
| L-serine ammonia-lyase, subunit beta | KQ693_07350 | 1000 | 886 | 1097 |

*Enzyme annotations originate from manual curation of RAST and GenBank (GCA_026427635.1) annotations.

**Ranks in the list of proteome proteins arranged according to their relative molar abundances (riBAQ values) in descending order. AutAer, AutAna, Hetero – the variants of cell growth: autotrophic aerobic, autotrophic anaerobic, heterotrophic (see main text).
