# Supplementary material for: Autotrophic growth of Thermus sp. PS18 and its genomic determinants shed light on the autotrophic lifestyle and its evolution in the Thermaceae family
Source: Front Microbiol. 2026 Mar 12;17:1769897. doi: 10.3389/fmicb.2026.1769897 (PMC13019369; doi:10.3389/fmicb.2026.1769897)
Supplement: Supplementary file 4 [file Table_4.docx]

**Supplementary Table 4.** Enzymes of the Sox system in *T. brevis* PS18

| **Enzyme*** | **GenBank**  **Locus tag** | **AutAer**  **rank**** | **Hetero**  **rank**** |
| --- | --- | --- | --- |
| Cytochrome c SoxD | KQ693_00400 | 248 | 1168 |
| sulfite dehydrogenase SoxC | KQ693_00405 | 36 | 644 |
| NAD(P)/FAD-dependent oxidoreductase | KQ693_00410 | 20 | 285 |
| Translation initiation factor 2 | KQ693_00415 | 5 | 126 |
| Rhodanese-like domain-containing protein | KQ693_00420 | 98 | 789 |
| Sulfur oxidation c-type cytochrome SoxA | KQ693_00425 | 28 | 507 |
| Sulfur oxidation c-type cytochrome SoxX | KQ693_00430 | 3 | 596 |
| Thiosulfohydrolase SoxB | KQ693_00435 | 56 | 645 |
| Sulfur oxidation c-type cytochrome SoxX | KQ693_00440 | 49 | 1001 |
| Sulfur oxidation c-type cytochrome SoxA | KQ693_00445 | 35 | 508 |
| Thiosulfate oxidation carrier protein SoxZ | KQ693_00450 | 6 | 123 |
| Thiosulfate oxidation carrier protein SoxY | KQ693_00455 | 8 | 170 |
| Thioredoxin family protein | KQ693_00460 | 168 | 1105 |

*Enzyme annotations originate from manual curation of RAST and GenBank (GCA_026427635.1) annotations.

**Ranks in the list of proteome proteins arranged according to their relative molar abundances (riBAQ values) in descending order. AutAer, Hetero – the variants of cell growth: autotrophic aerobic and heterotrophic (see main text).
