# Supplementary material for: Autotrophic growth of Thermus sp. PS18 and its genomic determinants shed light on the autotrophic lifestyle and its evolution in the Thermaceae family
Source: Front Microbiol. 2026 Mar 12;17:1769897. doi: 10.3389/fmicb.2026.1769897 (PMC13019369; doi:10.3389/fmicb.2026.1769897)
Supplement: Supplementary file 5 [file Table_5.docx]

**Supplementary Table 5.** Nitrate respiration enzymes in *T. brevis* PS18

| **Enzyme*** | **GenBank**  **Locus tag** | **TMHs** |
| --- | --- | --- |
| Hypothetical protein DrpB | KQ693_11285 | 1 |
| Hypothetical protein DrpA | KQ693_11290 | 3 |
| Respiratory nitrate reductase (EC 1.7.99.4) NarG subunit | KQ693_11330 | 0 |
| Respiratory nitrate reductase (EC 1.7.99.4) NarH subunit | KQ693_11325 | 0 |
| Respiratory nitrate reductase (EC 1.7.99.4) NarJ chaperone | KQ693_11320 | 0 |
| Respiratory nitrate reductase (EC 1.7.99.4) NarI subunit | KQ693_11315 | 5 |
| Nitrate/nitrite transporter NarK | KQ693_11310 | 12 |
| NarK2 Nitrate/nitrite transporter NarK | KQ693_11305 | 12 |
| *c-*type cytochrome NarC | KQ693_11335 | 1 |
| Hypothetical protein DnrT | KQ693_11340 | 0 |
| Transcriptional regulator DnrS, Crp/Fnr family | KQ693_11345 | 0 |
| Nitrite reductase (EC 1.7.2.1) NirS | KQ693_11385 | 1 |
| Nitric oxide reductase (EC 1.7.99.7*)* NorB subunt | KQ693_11395 | 12 |
| Nitric oxide reductase (EC 1.7.99.7) NorC subunit | KQ693_11400 | 2 |

*Enzyme annotations originate from manual curation of RAST and GenBank (GCA_026427635.1) annotations.

TMHs, number of transmembrane helices.
