# Supplementary material for: Autotrophic growth of Thermus sp. PS18 and its genomic determinants shed light on the autotrophic lifestyle and its evolution in the Thermaceae family
Source: Front Microbiol. 2026 Mar 12;17:1769897. doi: 10.3389/fmicb.2026.1769897 (PMC13019369; doi:10.3389/fmicb.2026.1769897)
Supplement: Supplementary file 6 [file Table_6.docx]

**Supplementary Table 6.** Formate dehydrogenase presumably involved in nitrate respiration in *T. brevis* PS18

| **Enzyme*** | **Locus tag** | **TMHs** |
| --- | --- | --- |
| Formate dehydrogenase O (EC 1.2.1.2) alpha subunit (FdoG) | KQ693_08000 | 1 |
| Formate dehydrogenase O (EC 1.2.1.2) beta subunit (FdoH) | KQ693_08005 | 1 |
| NrfD family protein | KQ693_08010 | 8 |
| Formate dehydrogenase accessory protein FdoE | KQ693_08015 | 0 |
| Formate dehydrogenase accessory protein FdoD | KQ693_08020 | 3 |

*Enzyme annotations originate from manual curation of RAST and GenBank (GCA_026427635.1) annotations.

TMHs, number of transmembrane helices.
