# Supplementary material for: Autotrophic growth of Thermus sp. PS18 and its genomic determinants shed light on the autotrophic lifestyle and its evolution in the Thermaceae family
Source: Front Microbiol. 2026 Mar 12;17:1769897. doi: 10.3389/fmicb.2026.1769897 (PMC13019369; doi:10.3389/fmicb.2026.1769897)
Supplement: Supplementary file 8 [file Data_Sheet_1.docx]

**Supplementary Text 1.** The origins of chemicals used in proteome analysis.

The following chemicals were used in proteome analysis: sequencing-grade modified trypsin (Promega); 2-chloroacetamide, formic acid, sodium deoxycholate, trifluoroacetic acid, Tris(2-carboxyethyl) phosphine hydrochloride, and Empore SPE disks SDB-RPS, SCX, and C18 (Sigma-Aldrich); Tris(hydroxymethyl)aminomethane (Panreac); ammonium acetate (Fluka); hypergrade-quality acetonitrile for LC-MS (LiChrosolv), acetone for liquid chromatography (LiChrosolv), gradient-grade methanol for liquid chromatography (LiChrosolv), and HPLC-grade water purchased from Merck (Darmstadt, Germany).

**Supplementary figures**


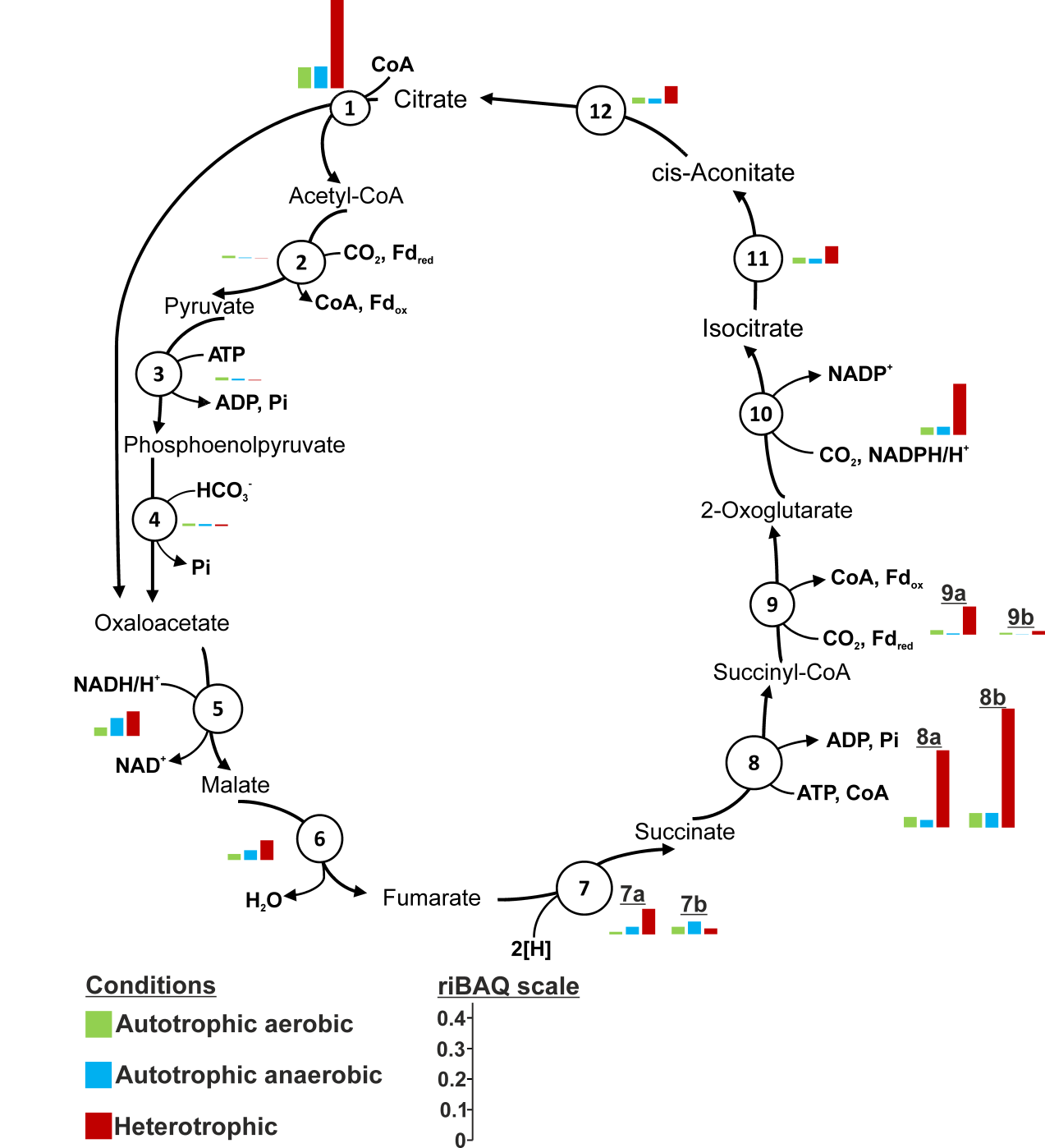


**Supplementary Figure 1.** Potential roTCA cycle in *T. brevis* PS18 according to the determinants found in the genome and the expression of these determinants as dependent on cultivation conditions and revealed by proteomic analysis. 1, citrate synthase (si) KQ693_06075; 2, pyruvate:ferredoxin (flavodoxin) oxidoreductase KQ693_09635; 3, phosphoenolpyruvate synthase KQ693_00705; 4, phosphoenolpyruvate carboxylase KQ693_09960; 5, malate dehydrogenase KQ693_06430; 6, fumarate hydratase class II KQ693_07060; 7, succinate dehydrogenase KQ693_12015, KQ693_12020; 8, succinyl-CoA ligase [ADP-forming] KQ693_06960, KQ693_06965; 9, 2-oxoglutarate:ferredoxin oxidoreductase KQ693_02940, KQ693_02935; 10, isocitrate dehydrogenase [NADP] KQ693_11820; 11, 12, aconitate hydratase KQ693_07290

**Supplementary Figure 2.** Potential reductive glycine pathway serine variant in *Thermus* sp. PS18 according to the determinants found in the genome and the expression of these determinants as dependent on cultivation conditions and revealed by proteomic analysis. **1,** KQ693_09560 - NAD-dependent formate dehydrogenase (KQ693_09560 - 09550); **2,** formate-tetrahydrofolate ligase (KQ693_03985); **3 and 4,** bifunctional 5,10-methylene-tetrahydrofolate dehydrogenase/5,10-methylene-tetrahydrofolate cyclohydrolase (KQ693_07895); **5,** glycine cleavage system (KQ693_06335 - 06350); **6,** serine hydroxymethyltransferase (KQ693_00830); 7, L-serine ammonia-lyase (KQ693_09900, KQ693_07350).

Glycine reductase complex is not encoded in the genome.


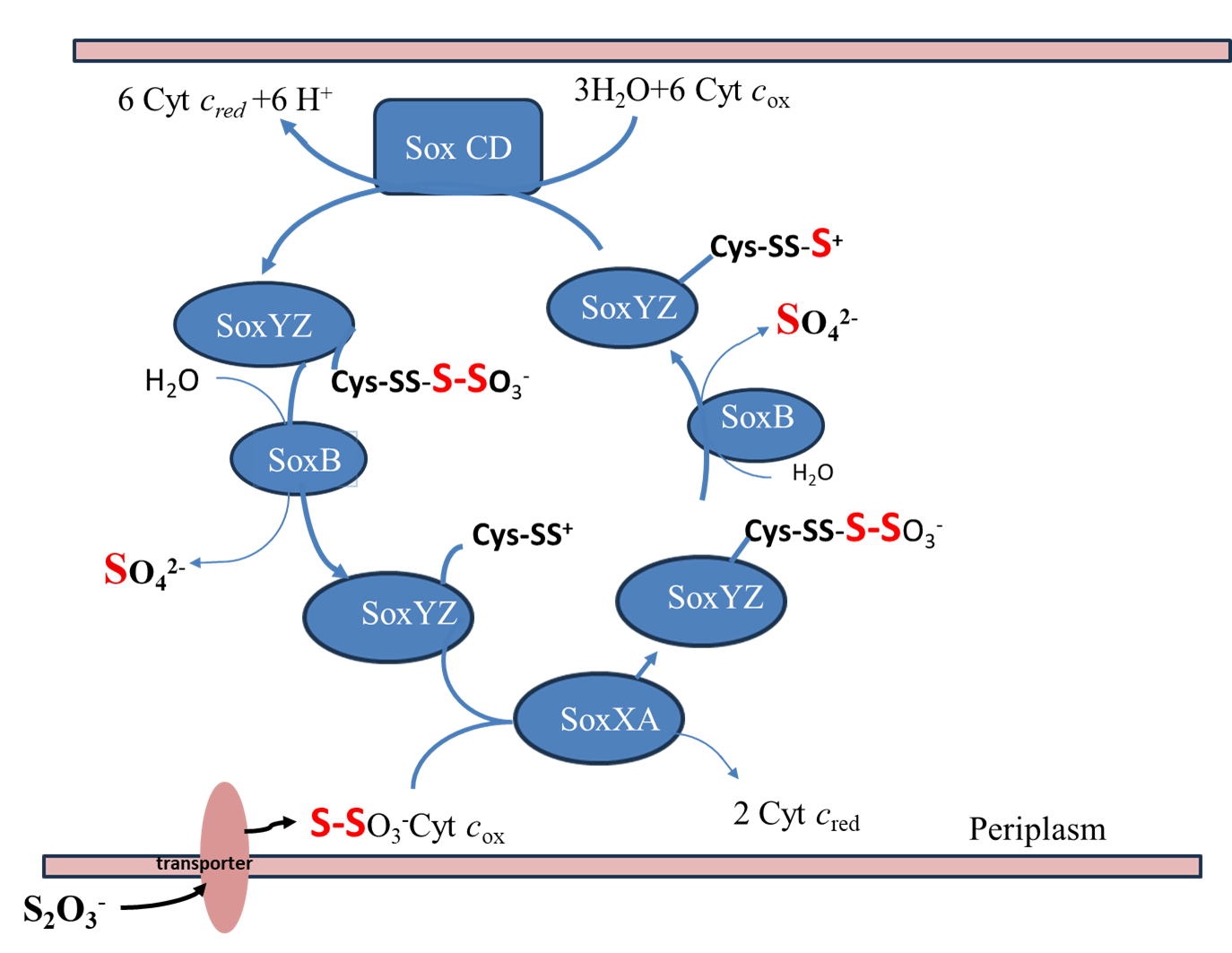


**Supplementary Figure 3.** Sox system of thiosulfate oxidation in *Thermus* sp. PS18 (based on the mechanism description by Sander and Dahl, 2009, <https://doi.org/10.1007/978-1-4020-8815-5_30>). **SoxA** - sulfur oxidation c-type cytochrome (KQ693_00445 and KQ693_00425); **SoxX** - sulfur oxidation c-type cytochrome (KQ693_00440 and KQ693_00430); **SoxY** - thiosulfate oxidation carrier protein (KQ693_00455); **SoxZ** - thiosulfate oxidation carrier protein (KQ693_00450); **SoxB** - thiosulfohydrolase (KQ693_00435); **SoxC** - sulfite dehydrogenase (KQ693_00405); **SoxD** - cytochrome c (KQ693_00400); transporter – sulfur compound transporter (KQ693_10565). In all of the Sox proteins of *Thermus* sp. PS18 except SoxZ, our analysis revealed signal peptides, whereas SoxZ was predicted by SecretomeP-2.0 to be a non-classically secreted protein.

**Supplementary Figure 4.** Enzymes of the Sox system in the proteomes of *Thermus* sp. PS18. **SoxZ,** thiosulfate oxidation carrier protein; **SoxY,** thiosulfate oxidation carrier protein; **SoxA1, s**ulfur oxidation *c*-type cytochrome; **SoxA2, s**ulfur oxidation c-type cytochrome; **SoxX1, s**ulfur oxidation c-type cytochrome; **SoxX2,** sulfur oxidation c-type cytochrome; **SoxC,** sulfite dehydrogenase; **SoxB,** thiosulfohydrolase; **SoxD,** cytochrome *c*.


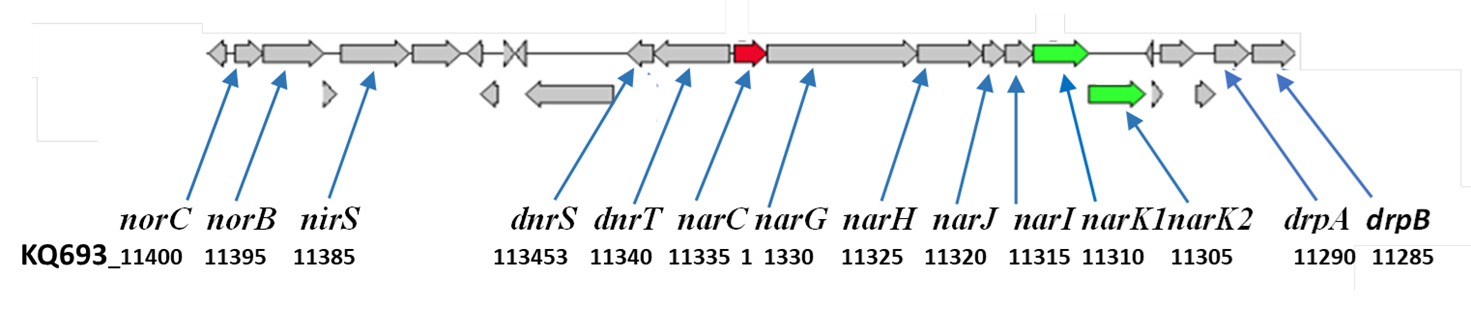
**Supplementary Figure 5.** Genomic determinants of nitrate respiration in *Thermus* sp. PS18. Gene names and GenBank locus tags are indicated for RAST/SEED visualization of the genome. See main text and Table S5 for more details.


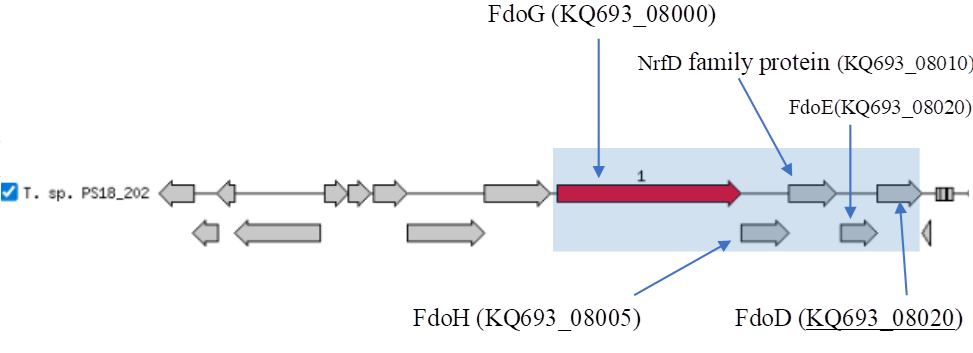


**Supplementary Figure 6.** Gene cluster encoding formate dehydrogenase O, involved in nitrate reduction by formate in *Thermus* sp. PS18. Names of encoded proteins and GenBank locus tags are indicated for RAST/SEED visualization of the genome. See main text and Table S4 for more details.

**Supplementary Figure 7.** Growth of *T. caldilimi* YIM 78456^T^ (triangles) and thiosulfate oxidation (circles) in mineral medium under aerobic conditions.


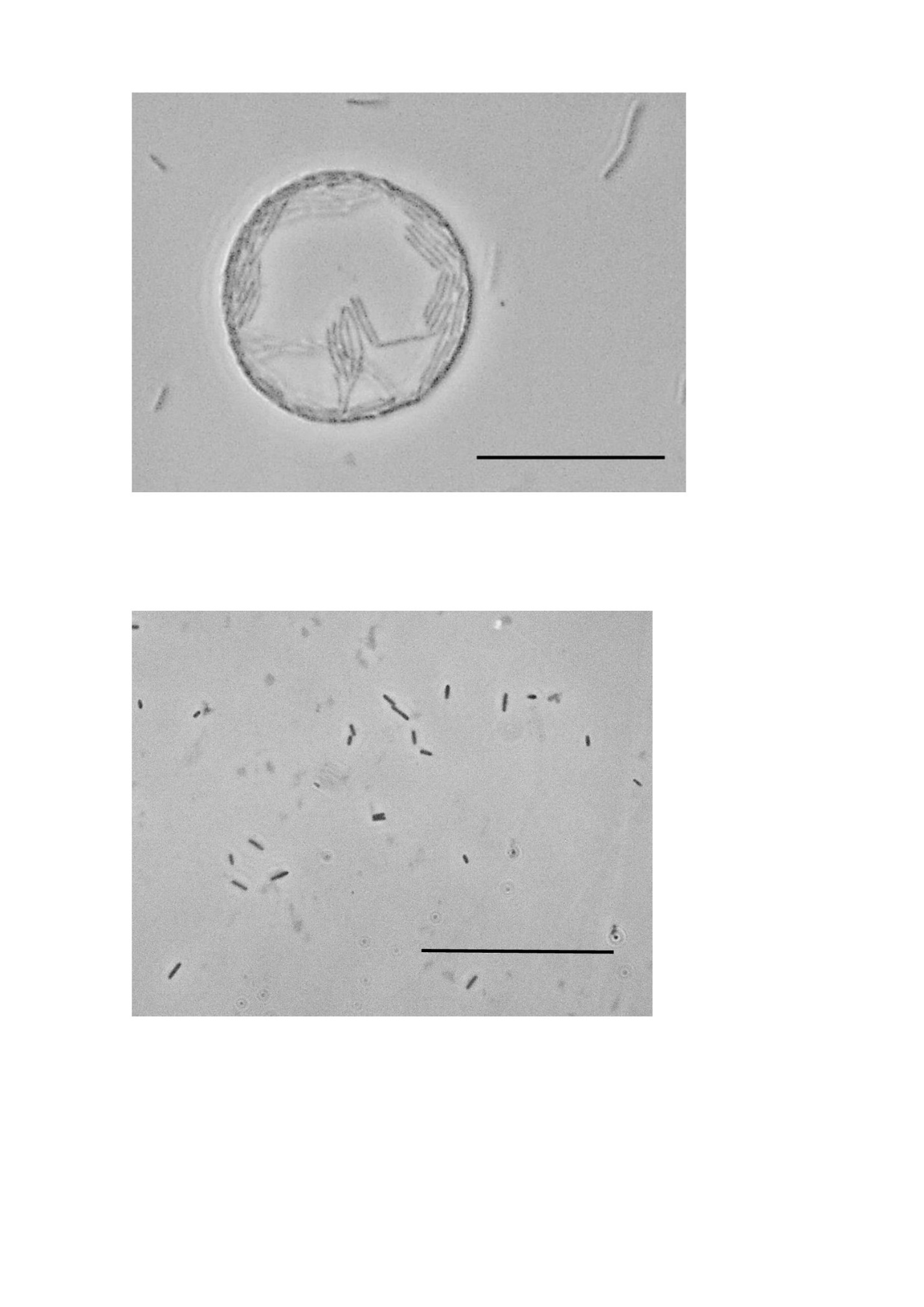


**Supplementary Figure 8.** Phase-contrast micrograph of autotrophically grown strain Uz8 cells, showing overall cell morphology. Bar, 10 µm


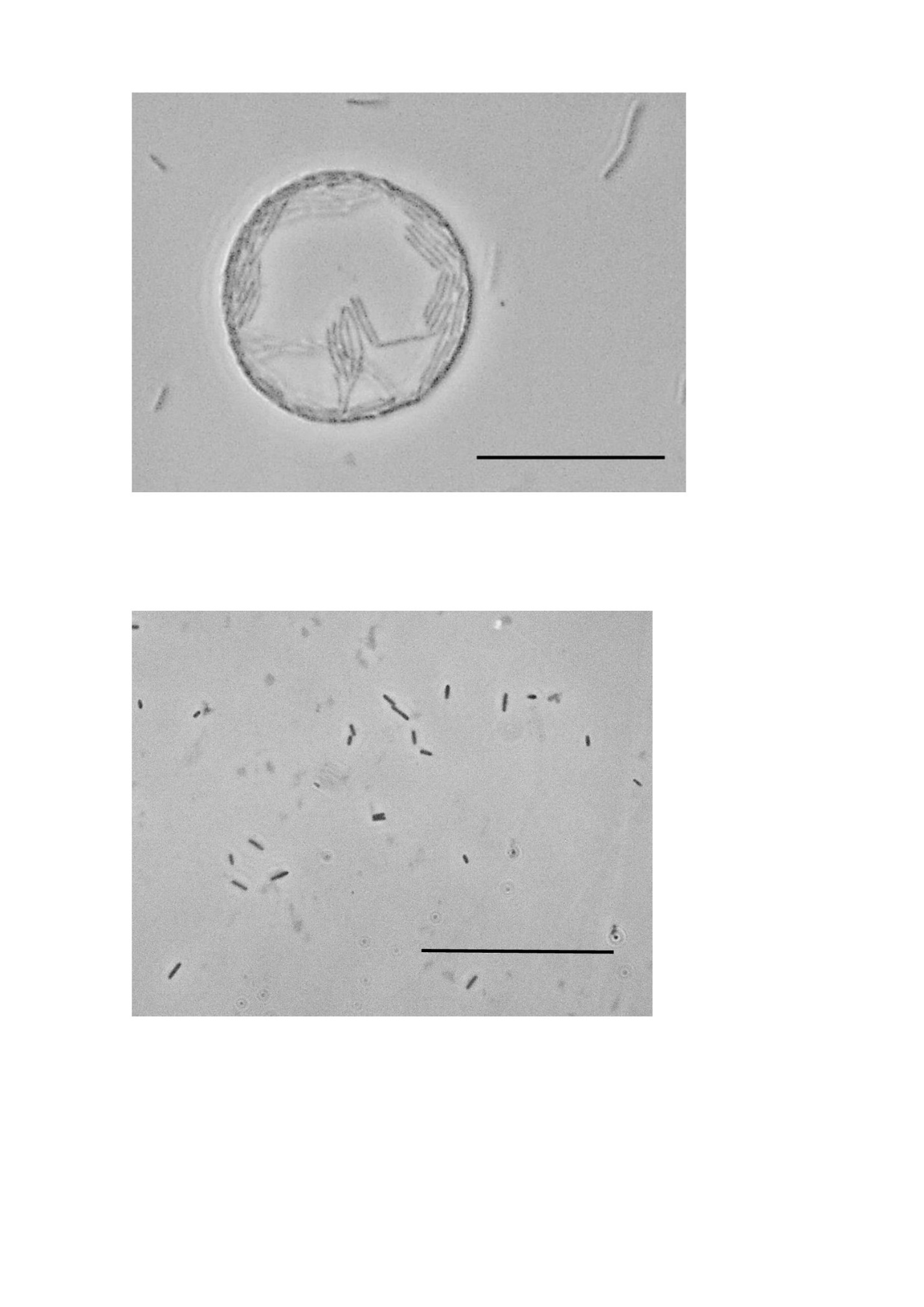


**Supplementary Figure 9.** Phase-contrast micrograph of herotrophically grown strain Uz79 cells, showing overall cell morphology and “rotund bodies”, characteristic for the species of the genus *Thermus*. . Bar, 10 µm.


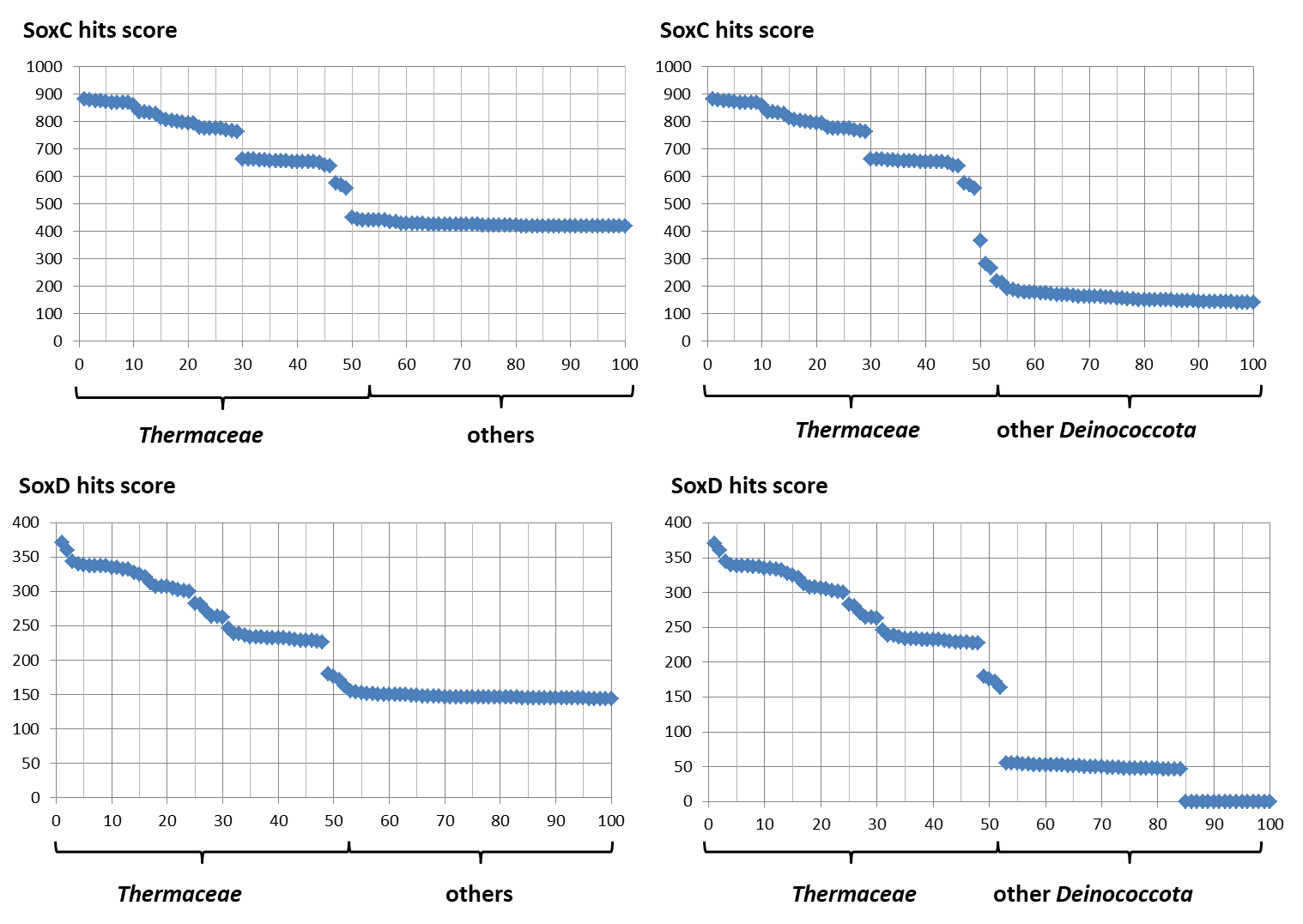


**Supplementary Figure 10.** tblastn of *Thermus* sp. PS18 SoxC and SoxD against all GTDB R220 representative genomes (left panels) and against GTDB R220 representative genomes of the phylum *Deinococcota* (right panels). 100 best hits are shown. The results demonstrate that closest homologs of *Thermaceae* *soxCD* genes are beyond the *Deinococcota* phylum.


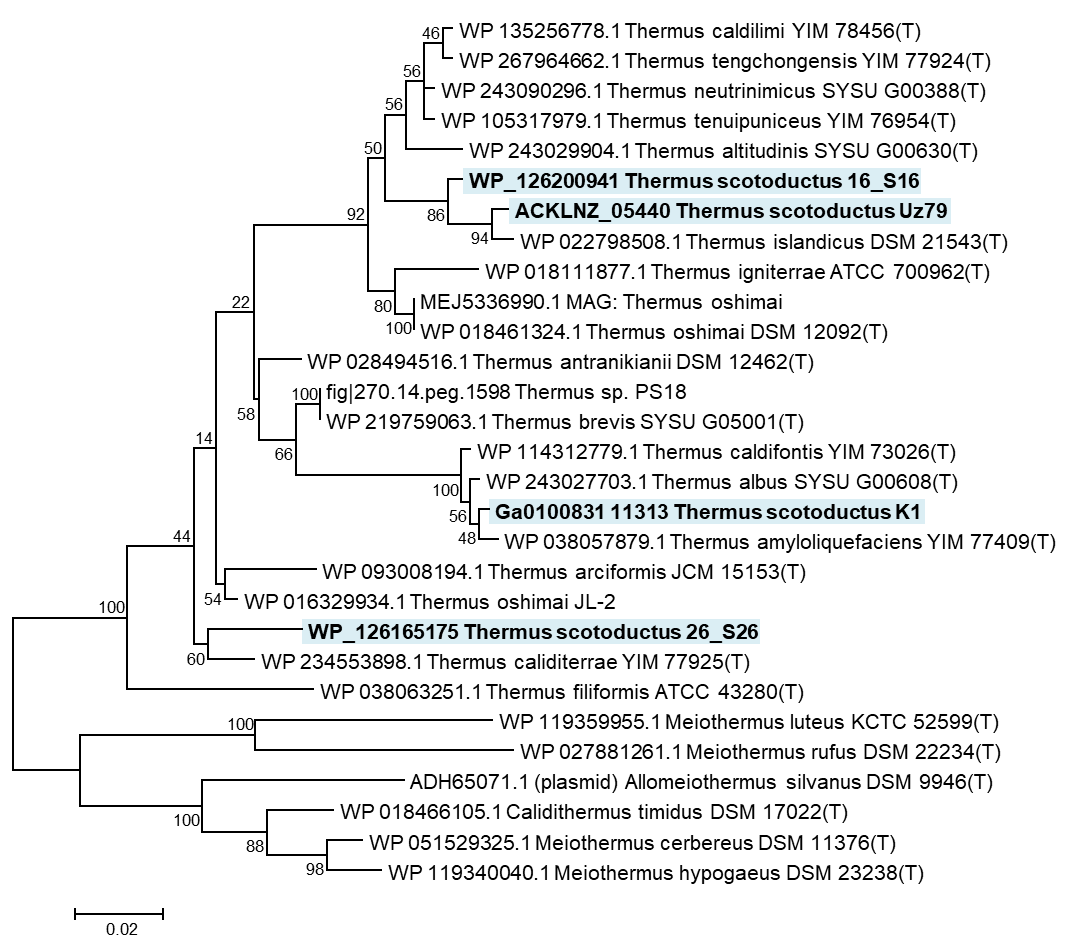


**Supplementary Figure 11.** Phylogenetic tree of RuBisCO large subunits from type strains of *Thermaceae* spp. and a few non-type strains of *Thermus* spp. Highlighted are *T. scotoductus* strains.
